# Bigger is not always better: Optimizing leaf area index with narrow-leaf shape in soybean

**DOI:** 10.1093/plphys/kiaf663

**Published:** 2025-12-26

**Authors:** Bishal G Tamang, Gregory Bernard, Carl J Bernacchi, Brian W Diers, Elizabeth A Ainsworth

**Affiliations:** Carl R. Woese Institute for Genomic Biology, University of Illinois, Urbana, IL 61801, United States; Department of Plant Biology, University of Illinois, Urbana, IL 61801, United States; Department of Plant and Soil Sciences, Tuskegee University, AL 36088, United States; Department of Crop Sciences, University of Illinois, Urbana, IL 61801, United States; Department of Crop Sciences, University of Illinois, Urbana, IL 61801, United States; Carl R. Woese Institute for Genomic Biology, University of Illinois, Urbana, IL 61801, United States; Department of Plant Biology, University of Illinois, Urbana, IL 61801, United States; Department of Crop Sciences, University of Illinois, Urbana, IL 61801, United States

## Abstract

Modern soybean (*Glycine max*) varieties have higher than optimal leaf area index (LAI), which could divert resources from reproductive growth. Altering leaf shape could be a simple strategy to reduce LAI. To test this, we developed 204 near-isogenic lines differing in leaf morphology by introgressing the *ln* allele that confers the narrow-leaf trait from 2 donor parents (PI 612713A and PI 547745) into the elite, broad-leaf cultivar LD11-2170. We evaluated the lines across 2 locations and 2 row spacings (38 cm and 76 cm) to assess how reduced investment in leaf area influences canopy architecture, crop physiology, and yield. Narrow-leaf lines showed 13% lower peak LAI and 3% lower digital biomass compared to broad-leaf counterparts yet maintained yield parity (5,756 vs. 5,801 kg ha^−1^, *P* = 0.43) across environmental conditions. Photosynthetic capacity remained largely unchanged, with narrow-leaf lines showing modest increases in electron transport rate and leaf mass per area. Narrow-leaf lines achieved similar canopy closure timing despite lower LAI, suggesting architectural compensation mechanisms. The most striking difference appeared in seed packaging, with 34% of pods containing 4 seeds in narrow-leaf lines compared to only 1.8% in broad-leaf lines. There was a nonlinear relationship between peak LAI and yield, with optimal LAI values of 9 to 11 varying by environment. These findings show that the single-gene *GmJAG1*-controlled narrow-leaf trait offers a tractable strategy for reducing LAI and maintaining high productivity. This could reduce the metabolic costs associated with excessive canopy development and support sustainable agriculture under increasing climate variability.

## Teaser

Narrow-leaf shape in soybean allows plants to achieve comparable yields with reduced canopy size, offering breeders a simple tool for developing more resource use efficient varieties.

## Introduction

Soybean (*Glycine max* (L.) Merr.) provides a critical source of protein, oil, and bioactive compounds for human consumption, animal feed, and industrial applications (Messina 2022; soystats.com: accessed on 21 April 2025). Over the past few decades, global soybean production has expanded dramatically to meet increasing demand (FAO 2023), but future production must address the dual challenges of climate change and limited arable land expansion (Lu et al. 2021; Goulart et al. 2023). This necessitates developing varieties with improved resource use efficiency to maintain and enhance productivity without increasing environmental footprints, a priority explicitly highlighted in the Soybean Genomic Research Community Strategic Plan (2024–2028), which underscores the need for innovative breeding strategies to develop resource-efficient, climate resilient cultivars (Stupar et al. 2024).

Agricultural sustainability increasingly depends on optimizing crop architecture for maximal productivity per unit of resource invested. The Green Revolution increased yields through architectural modifications, including reduced plant height and altered leaf angles to improve light interception and input responsiveness (Sakamoto and Matsuoka 2004). Today's “ideotype breeding” approach designs plants with ideal architectural and physiological traits for specific environments and cropping systems. Within this framework, leaf morphology in soybean represents a fundamental yet underexplored component of plant architecture with significant potential for optimizing resource allocation and enhancing yield potential (Roth et al. 2022; Clark and Ma 2023; Li et al. 2024).

Plant architecture profoundly influences crop performance by determining spatial patterns of light interception, gas exchange, and resource allocation (Nobel et al. 1993; Croce et al. 2024). In field conditions, individual plants function collectively as a canopy, where architectural traits dictate the efficiency of capturing available light energy for photosynthesis. For instance, the vertical distribution of leaf area and leaf angle controls light penetration, with upright leaves allowing greater illumination of lower canopy layers and enhancing whole-canopy photosynthesis (Sarlikioti et al. 2011; Mantilla-Perez and Fernandez 2017; Yang et al. 2023; Sreekanta et al. 2024). Architectural traits also shape microclimates which affect water use efficiency, and they orchestrate nutrient utilization through distribution of photosynthetic capacity and nitrogen allocation within the canopy (Moreau et al. 2012). Further, modern crop modeling approaches increasingly incorporate detailed features of canopy architecture to capture these complex interactions. As climate variability increases, architectural traits that optimize physiological processes will become even more critical for maintaining crop production (Long et al. 2015; Liu et al. 2021).

Leaf area index (LAI), defined as the ratio of leaf area to ground area, serves as a critical indicator of canopy development and potential productivity (Parker 2020; Wei et al. 2023). Optimal LAI varies by crop species, environment, and management, with both insufficient and excessive values of LAI potentially reducing yield. Insufficient LAI reduces light interception and photosynthetic capacity, while excessive LAI can lead to self-shading, reduced light use efficiency, and unnecessary allocation of resources to vegetative growth at the expense of reproductive development (Digrado and Ainsworth 2023). For soybeans specifically, studies have identified nonlinear relationships between LAI and yield, with productivity peaking at optimal LAI values ranging from 3.5 to 6.5 depending on cultivar and growing conditions (Board and Harville 1992; Liu et al. 2008; Setiyono et al. 2008; Tagliapietra et al. 2018). Research by Srinivasan et al. (2017) combining modeling and empirical approaches demonstrated that modern soybean varieties overproduce leaf area, a condition likely to worsen under future elevated atmospheric CO_2_ concentrations that increase biomass production (Ainsworth and Long 2005; Soares et al. 2021). This nonlinear relationship suggests that maximizing leaf area may not be the optimal strategy for enhancing yield, particularly when considering the metabolic costs of leaf production and maintenance.

Plants face fundamental trade-offs in allocating limited resources between vegetative and reproductive structures. Leaves represent significant investment of carbon, nitrogen, and other nutrients, which must be balanced against investments in stems, roots, and reproductive organs (Holland et al. 2019). Leaf morphology—including size, shape, thickness, and arrangement—directly influences this resource allocation equation, affecting both the cost of leaf construction and the return on investment through photosynthetic output (Onoda et al. 2017; Villar et al. 2021). In crops, leaf shape diversity reflects both natural and breeding efforts. Breeding programs have traditionally focused on maximizing yield, which has indirectly increased leaf area. This approach may not optimize overall resource use efficiency. Narrow leaves potentially offer advantages in terms of reduced canopy structural investment, improved light penetration through the canopy and leaf-level photosynthetic rates (Tamang et al. 2023), which affects nitrogen distribution efficiency and photosynthetic nitrogen use efficiency (Qiang et al. 2023). Similar morphological modifications have been shown to enhance photosynthesis in rice (*Oryza sativa*), where natural variation in the *NARROW LEAF1* (*NAL1*) gene resulted in narrower, thicker leaves with increased mesophyll cell density and ∼30% higher photosynthetic rates (Takai et al. 2013).

Recent advances in molecular genetics have identified key regulators of leaf morphology in soybean. The *ln* (narrow leaf) locus, encoding the JAGGED-like transcription factor *GmJAG1* (Glyma.20G116200; Wm82.a2.v1), has emerged as a critical determinant of leaflet shape. A single nucleotide polymorphism (SNP) within the *ETHYLENE-RESPONSIVE ELEMENT BINDING FACTOR-ASSOCIATED AMPHIPHILIC REPRESSION* (EAR) motif of *GmJAG1* causes an amino acid substitution (aspartic acid to histidine), disrupting its repressor function and resulting in a narrow-leaf phenotype (Jeong et al. 2012). In *Arabidopsis thaliana*, the homologous *AtJAG1* promotes organ growth by repressing KIP-RELATED PROTEIN (KRP) genes, which inhibit the DNA synthesis phase of the cell cycle, thereby enhancing cell division (Schiessl et al. 2014). In soybean, *GmJAG1* likely operates similarly, with the nonfunctional allele reducing leaflet width by limiting cell proliferation, leading to elongated, narrower leaves. These morphological changes alter canopy architecture by reducing LAI and enhancing light penetration, potentially improving photosynthetic efficiency and resource allocation (Tamang et al. 2023).

Building on this genetic insight, our study leverages *GmJAG1's* role to explore leaf shape's broader impacts, addressing gaps in prior research. Despite some advances in understanding soybean leaf shape effects on canopy development and yield, existing studies have been limited in scale and genetic diversity. For instance, Bianchi et al. (2020) found narrow leaves improved light penetration but used only 4 isogenic lines, limiting statistical power and generalizability. Similarly, earlier work by Mandl and Buss (1981) and Wells et al. (1993) faced comparable limitations in genetic material. More recently, Cai et al. (2021) demonstrated yield benefits of narrow-leaf soybeans through a gene editing approach, yet overlooked canopy dynamics. Our study overcomes these constraints by investigating over 200 near-isogenic lines that differ primarily in leaf shape, providing statistical power and genetic diversity to examine the relationships between leaf shape, LAI, and productivity. Understanding these relationships provides valuable insights for breeding programs seeking to enhance resource use efficiency and yield stability in soybean in future climatic conditions.

The objectives of this study were to (i) characterize the effects of leaf shape on canopy development dynamics, including LAI, canopy coverage and biomass accumulation; (ii) assess whether altered leaf morphology affects photosynthetic capacity at leaf level; (iii) determine the impact of leaf shape on yield components and seed quality parameters; (iv) investigate how environmental conditions and management practices interact with leaf shape to influence plant performance; and (v) identify key traits and processes mediating the relationship between leaf morphology and yield. To achieve these objectives, we developed and evaluated 204 near-isogenic soybean lines with a wide range of leaf shape across 2 environments and 2 management practices. Our phenotyping approach integrated detailed leaf-level measurements with canopy-scale assessments and yield evaluations, providing a holistic understanding of how leaf shape influences the source-sink continuum from photosynthetic capacity to yield.

## Results

### Isogenic lines differ in leaf shape, not phenology

**Figure 1.**
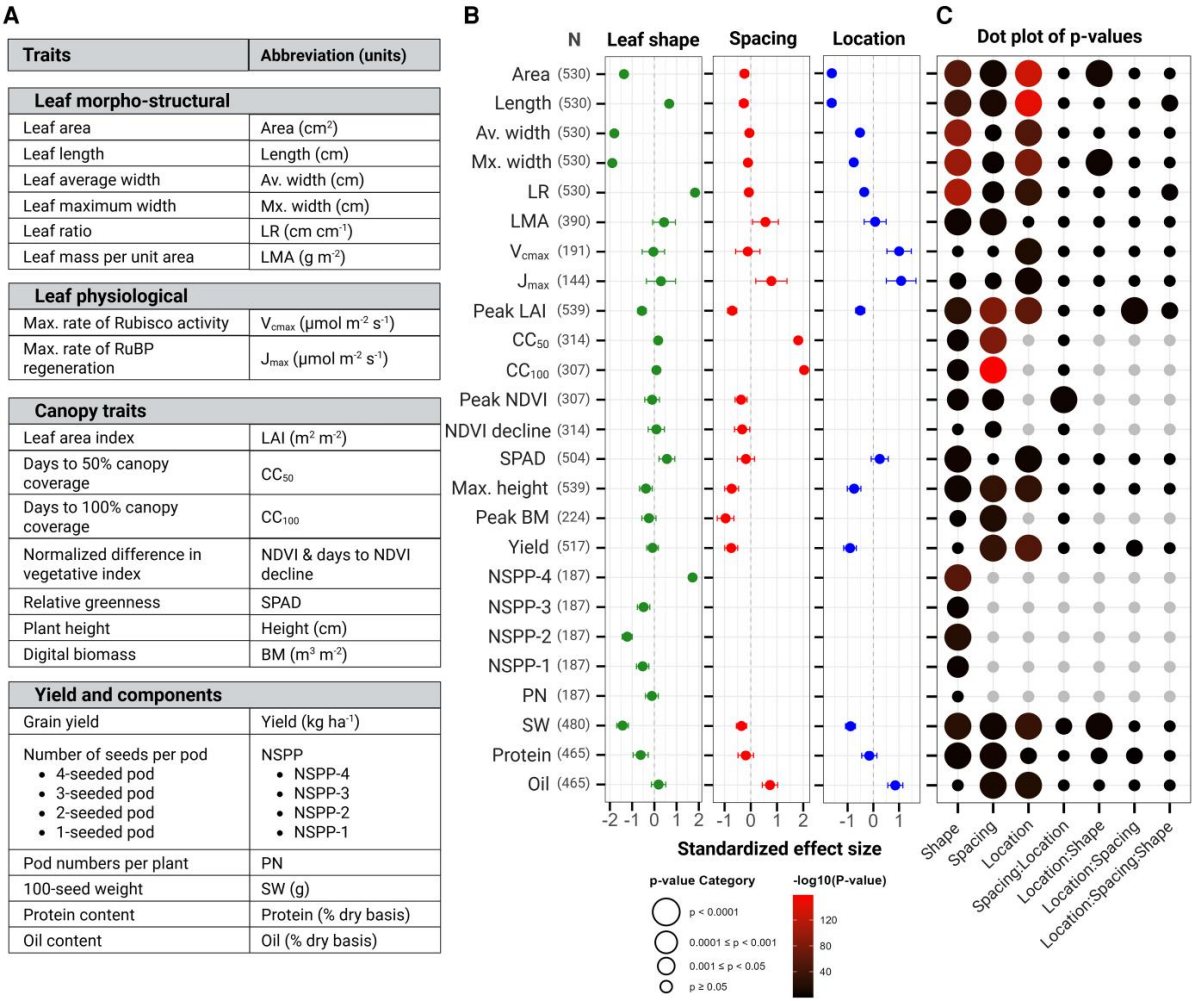
Mixed modeling results for trait responses to experimental factors. **A)** List of measured traits with their corresponding abbreviations and units of measurement. **B)** Standardized effect size (±95% confidence intervals) from mixed models for each trait in response to leaf shape (representing effect of narrow leaf relative to broad-leaf shape as reference, left panel), spacing (representing effect of 76-cm spacing relative to 38-cm spacing as reference, middle panel), and location (representing effect of location SoyFACE relative to Energy Farm as reference, right panel). The *y* axis displays trait abbreviations with sample sizes (*N*) used in the mixed models for each trait. **C)** Significance of main effects and interactions from mixed models. The *x* axis displays the 3 main experimental factors (leaf shape, spacing, and location) and their 4 interaction terms (leaf shape × spacing, leaf shape × location, spacing × location, and leaf shape × spacing × location). Circle size represents significance categories of *P*-values, while circle color intensity corresponds to -log_10_(*P*-value), with brighter colors indicating higher statistical significance. All effects are relative to the respective reference levels (broad leaf shape, 38-cm spacing, and location Energy Farm). Positive effect sizes indicate that narrow leaves, 76-cm spacing, or SoyFACE location increased the trait value relative to their respective reference levels (broad leaves, 38-cm spacing, or Energy Farm location), while negative effect sizes indicate a decrease.

**Figure 2.**
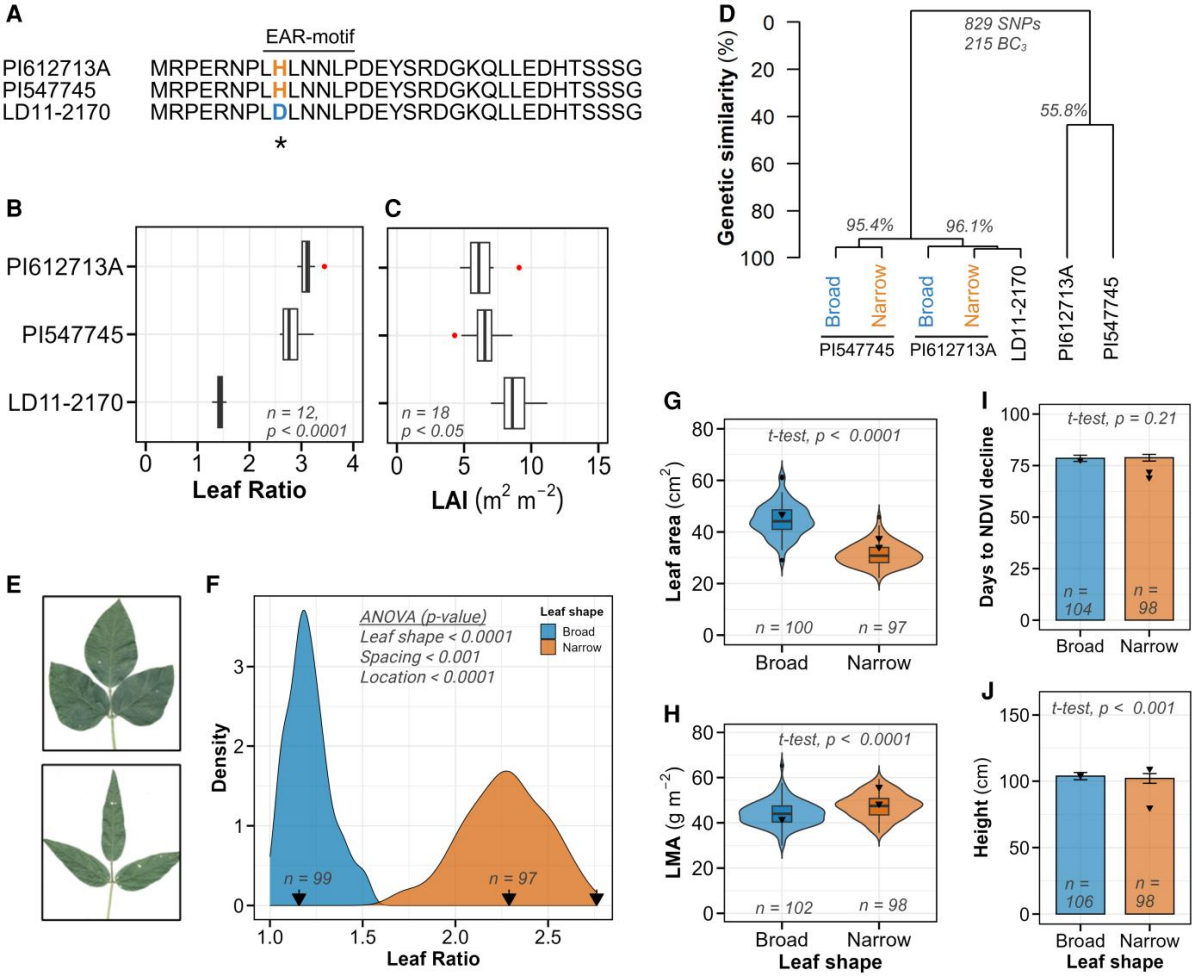
Molecular characterization and phenotypic differences of parental and isogenic lines. **A)** Amino acid sequence alignment of GmJAG1 from 3 parental lines: recurrent parent LD11-2170 and narrow-leaf donor parents PI 612713A and PI 547745. The sequence displays the first 33 amino acids from the start codon (M), highlighting the critical SNP that causes an amino acid substitution from aspartic acid (D, blue) in LD11-2170 to histidine (H, orange) in both narrow-leaf parents within the EAR motif region (indicated above the sequence) rendering the gene nonfunctional. b and c Box plots comparing leaf ratio **B)** and LAI **C)** among the 3 parental lines. **D)** Hierarchical clustering showing genetic similarity (%, *y* axis) among the 3 parental lines and 204 BC_3_ progeny based on 829 polymorphic SNP markers. The BC_3_ progenies were classified into broad and narrow-leaf categories derived from PI 547745 and PI 612713A, with percentage similarity values indicated at major branch points. **E)** Representative images of soybean trifoliate leaves from broad-leaf (upper panel) and narrow-leaf (lower panel) isogenic lines. **F)** Density plot of leaf ratio distribution for broad (blue) and narrow (orange) leaf phenotypes. Black arrows along the *x* axis indicate the 3 parental values. Sample sizes (*n*) for each leaf shape category and significant mixed model ANOVA *P*-values are provided within the panel. g and h) Violin plots with embedded box and whisker plots comparing leaf area **G)** and leaf mass per area **H)** between broad and narrow-leaf categories. Black triangles represent parental values, with sample sizes (*n*) and *t*-test *P*-values indicated. In boxplots: center line, median; box limits, 25th to 75th percentiles; whiskers, 1.5 × interquartile range. i and j) Bar plots showing days to NDVI decline **I)** and plant height **J)** between broad and narrow-leaf categories. Black triangles indicate parental values, with sample sizes (*n*) and *t*-test *P*-values provided for each comparison. In boxplots: center line, median; box limits, 25th to 75th percentiles; whiskers, 1.5 × interquartile range; points, outliers.

Using marker-assisted repeated backcrossing, we developed 204 isogenic lines that significantly differed in leaf shape and LAI (*P* < 0.0001), but not most developmental or physiological traits (Fig. 1A and B, Fig. S3). Genotyping with 1327 Agriplex SNPs (829 polymorphic between parents) confirmed that the isogenic lines shared between 92% and 99% genetic similarity with the broad-leaf recurrent parent LD11-2170 (Fig. 2D), close to the theoretical similarity of 93.95% expected after 3 backcrosses. A *GmJAG1*-specific KASP marker effectively discriminated between homozygous broad-leaf, heterozygous, and homozygous narrow-leaf lines (Figure S1). Sequencing confirmed that both PI 612713A and PI 547745 carry the identical *ln* allele, with the same G→C substitution causing the Asp→His amino acid change in the EAR motif (Fig. 2A).

The donor narrow-leaf parents (PI 612713A and PI 547745) exhibited distinct leaf morphology compared to the recurrent parent (LD11-2170), with approximately 2-fold higher leaf ratios (LR: 3.1 and 2.8 vs. 1.4, respectively) and substantially lower LAI (6.3 and 6.5 vs. 8.8, *P* < 0.05; Fig. 2B and C). The parents also differed significantly in LMA, height, V_cmax_, and J_max_ (*P* < 0.001; Figure S2a to e).

As intended, the resulting BC_3_ isogenic lines displayed a clear bimodal distribution in leaf ratio, with means of 1.3 and 2.4 for broad and narrow categories, respectively (Fig. 2E and F). This leaf shape variation showed a large effect size (1.3, *P* < 0.0001; Fig. 1B and C), indicating strong genetic control of this trait. The narrow-leaf phenotype was also associated with approximately 35% less leaf area (*P* < 0.0001) and 5% higher LMA (*P* < 0.0001) compared to broad-leaf lines (Fig. 2G and H). Location also significantly influenced leaf ratio (effect size = −0.35, *P* < 0.0001), while row spacing did not (*P* = 0.16).

Despite these morphological differences, both leaf types showed similar developmental progression. Days to NDVI decline (a proxy for reproductive maturity) averaged 79 d for both leaf types (*P* = 0.21; Fig. 2I), showing only marginal effects of spacing (effect size = 0.33, *P* = 0.026; Fig. 1B and C). Similarly, plant height differences between leaf types were statistically significant but minimal in magnitude (108.3 cm for broad vs. 106.3 cm for narrow, *P* < 0.01; Fig. 2J). These results confirm that our genetic intervention successfully altered leaf shape while maintaining comparable growth and development patterns, providing an ideal system to isolate the effects of leaf shape on canopy development and yield.

### Photosynthetic capacity is primarily determined by location, with minor influences from leaf morphology and row spacing

To investigate whether altered leaf morphology affected photosynthetic capacity, we analyzed CO_2_ response curves from 264 leaf samples representing all treatment combinations (Figure S4). Both maximum carboxylation rate (V_cmax_) and maximum electron transport rate (J_max_) varied strongly between locations, but did not vary as much by leaf shape or row spacing (Fig. 1B and C). This suggested that photosynthetic biochemistry was not substantially altered by the changes in leaf morphology.

**Figure 3.**
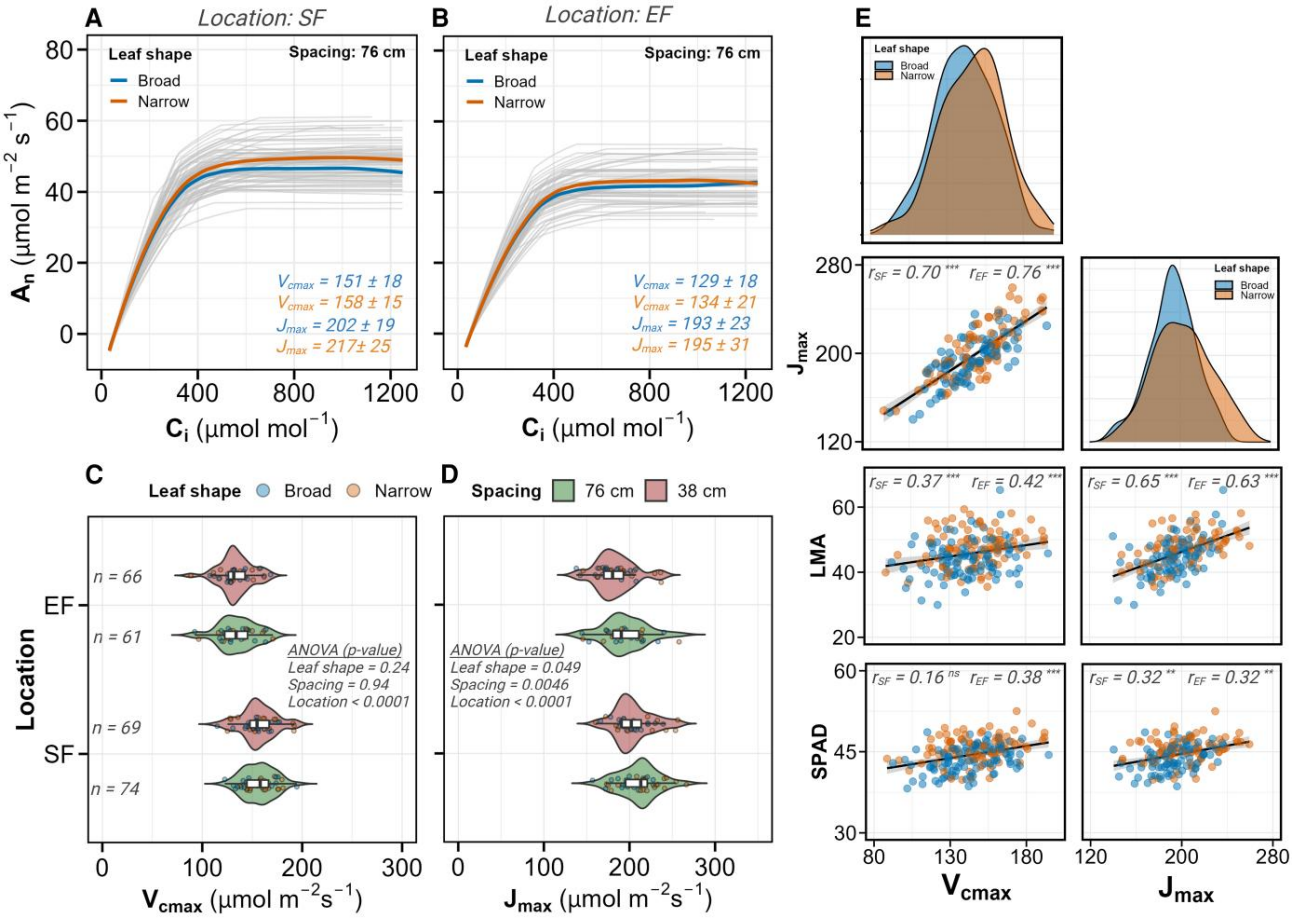
Photosynthetic parameters derived from A-C_i_ curve analysis and their correlation with leaf structure. **A** and **B)** Fitted A-C_i_ response curves from PhotoGEA R package for plants grown in 76-cm spacing at location SoyFACE (SF) a) and Energy Farm (EF) **B)**. Curves represent model outputs from the package, which were generated at 1 µmol mol^−1^ C_i_ intervals based on fitted parameters derived from measured data points. Individual fitted curves are shown in light gray, with average curves for broad (blue) and narrow (orange) leaf shapes highlighted. Mean values (±SD) for maximum carboxylation rate (V_cmax_) and maximum electron transport rate (J_max_) are displayed for each leaf shape with corresponding colors. **C** and **D)** Violin plots with embedded box and whisker plots showing V_cmax_  **C)** and J_max_  **D)** distribution across locations (SF and EF) and row spacings (76 cm in green, 38 cm in red). Individual data points are colored by leaf shape (broad in blue, narrow in orange). Sample size (*n*) and ANOVA *P*-values from mixed models are provided. **E)** Pairwise correlation analysis between photosynthetic parameters (V_cmax_, column 1; J_max_, column 2) and leaf traits measured from the same leaf. The top panel shows density distributions by leaf shape for the 2 photosynthetic parameters. Remaining panels display scatter plots with regression line (black) and 95% confidence intervals (shaded areas) for relationships between the photosynthetic parameters and leaf mass per area (LMA) and SPAD. Data points are colored by leaf shape (broad in blue, narrow in orange). Pearson correlation coefficients (*r*) are provided for each location (SoyFACE and Energy Farm) with significance indicated by asterisks (*P < 0.05, **P < 0.01, ***P < 0.001). In boxplots: center line, median; box limits, 25th to 75th percentiles; whiskers, 1.5 × interquartile range; points, outliers.

Location was the dominant factor affecting photosynthetic parameters, with large and significant effect size (1.0 to 1.1, *P* < 0.0001; Fig. 1B). Both V_cmax_ and J_max_ were substantially higher at SoyFACE compared to the Energy Farm (approximately 17% and 12% higher, respectively; Fig. 3A to D), likely reflecting differences in soil properties and/or microclimate between the 2 locations. These location-based differences were consistent across both leaf types and row spacings, underscoring the strong influence of environmental factors on photosynthetic capacity.

In contrast to the location effect on photosynthetic capacity, leaf shape had no significant influence on V_cmax_ (*P* > 0.2) and a small yet significant effect on J_max_ (effect size = 0.29, *P* < 0.05; Figs. 1B and 3A to D). Narrow-leaf isogenic lines had ∼4% higher J_max_ compared to their broad-leaf counterparts, suggesting a slight enhancement in electron transport capacity. Similarly, row spacing showed no significant effect on V_cmax_ but modestly influenced J_max_ (effect size = 0.78, *P* < 0.05), with 76-cm spacing associated with 6% higher J_max_ compared to 38-cm spacing.

Correlation analysis revealed strong positive associations between V_cmax_ and J_max_ across both locations (*r* = 0.70 to 0.76, *P* < 0.0001; Fig. 3E), consistent with the coordinated regulation of these biochemical traits. LMA showed a strong association with both V_cmax_ and J_max_ (*r* = 0.50 to 0.65, *P* < 0.0001). There was a weaker, but still significant correlation between SPAD, V_cmax_ and J_max_ (*r* = 0.16 to 0.38, *P* < 0.0001), reflecting the established link between leaf nitrogen allocation and photosynthetic capacity.

### Narrow-leaf morphology reduces LAI, with effects modulated by row spacing and location

**Figure 4.**
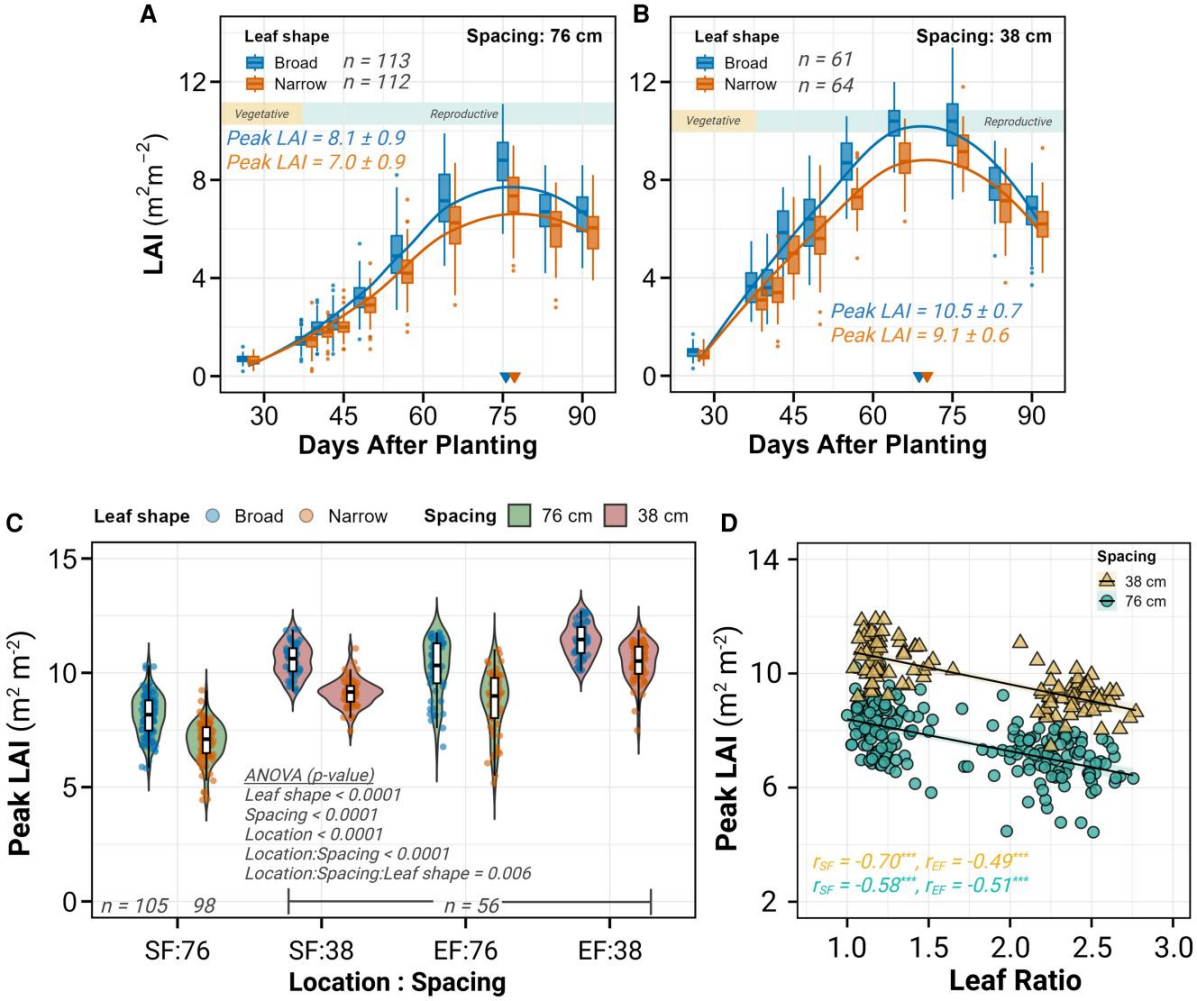
Temporal dynamics and association of LAI. **A** and **B)** Time course of LAI measurements at SoyFACE under 76-cm **A)** and 38-cm **B)** row spacing. Box and whisker plots display LAI evolution at ten time points for broad (blue) and narrow (orange) leaf shapes, with fitted growth curves in corresponding colors. The horizontal rectangle at the top indicates vegetative and reproductive growth stages. Colored triangles on the *x* axis mark the timing of peak LAI for each leaf shape. Sample sizes (*n*) and peak LAI values (±SD) are provided for both leaf shapes. **C)** Violin plots with embedded box and whisker plots comparing peak LAI across all location–spacing combinations. Plots are colored by row spacing (76 cm in green, 38 cm in red), with individual data points colored by leaf shape (broad in blue, narrow in orange). Sample sizes (*n*) and ANOVA *P*-values from mixed model analysis are displayed. **D)** Scatter plot showing the relationship between leaf ratio (*x* axis) and peak LAI (*y* axis) for location SF. Data points are differentiated by row spacing: 38 cm (buff triangles) and 76 cm (teal circles), with corresponding regression lines and shaded 95% confidence intervals. Pearson correlation coefficients (*r*) are provided for each location (SF, EF) and spacing treatment with significance indicated by asterisks (*P < 0.05, **P < 0.01, ***P < 0.001). In boxplots: center line, median; box limits, 25th – 75th percentiles; whiskers, 1.5 × interquartile range; points, outliers.

Narrow-leaf morphology consistently reduced canopy-level LAI across all treatment combinations, though the magnitude of this effect varied with both row spacing and location. By tracking the progression of LAI across the growing season (Fig. 4A and B; Figure S5a and b), we identified distinct patterns in canopy dynamics influenced by all 3 experimental factors.

LAI increased progressively from early vegetative stages (25 d after planting) until reaching maximum values during mid-reproductive development. Peak LAI occurred a week earlier in 38-cm rows (approximately 68 d after planting) than in 76-cm rows (approximately 75 d after planting) across both locations (Fig. 4A, B, Figure S5a, b). Within each spacing treatment, narrow-leaf lines reached peak LAI slightly later than broad-leaf lines (1.5 d later at SoyFACE and 2.5 d later at Energy Farm).

All 3 experimental factors significantly affected peak LAI with comparable effect sizes (0.51 to 0.70, *P* < 0.0001; Fig. 1B and C), indicating their similar importance in determining canopy development. Location had a substantial effect, with Energy Farm plots achieving 16% higher peak LAI than SoyFACE plots (10.2 vs. 8.59, *P* < 0.0001). Row spacing exerted an even stronger influence. The 38-cm spacing rows had 20% higher peak LAI compared to 76-cm spacing rows (10.39 vs. 8.5, *P* < 0.0001).

Most importantly for our research question, leaf morphology consistently affected peak LAI, with narrow-leaf lines showing 13.3% lower values compared to broad-leaf lines (8.8 vs. 10.1, *P* < 0.0001; Fig. 4A to C; Figure S6a and b). This reduction ranged from 8.7% to 15.3% across the 4 location–spacing combinations, indicating the consistency of this effect despite environmental and management differences. The negative association between leaf shape and canopy development was further supported by significant negative correlations between leaf ratio and peak LAI across all treatments (*r* = −0.49 to −0.70, *P* < 0.0001; Fig. 4D).

### Row spacing drives canopy coverage dynamics while leaf shape has minimal impact

**Figure 5.**
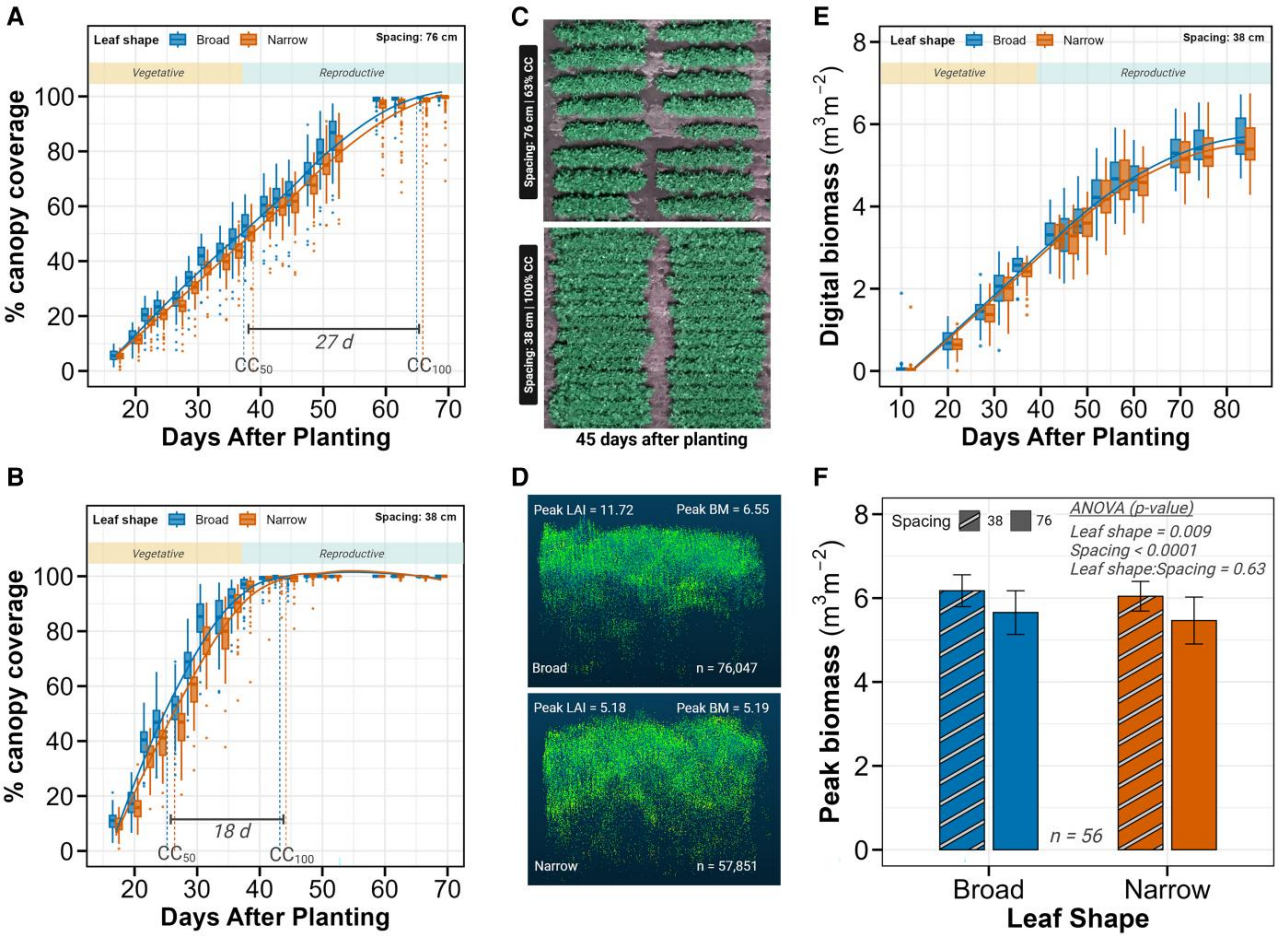
Canopy development dynamics and biomass accumulation across leaf types and row spacings. **A** and **B)** Temporal progression of percent canopy coverage at SoyFACE for 76-cm **A)** and 38-cm **B)** row spacing. Box and whisker plots show distributions at 20 time points for broad (blue) and narrow (orange) leaf shapes, with fitted curves in corresponding colors. The horizontal rectangle indicates vegetative and reproductive growth stages. Vertical colored lines mark the days to reach 50% (CC_50_) and 100% (CC_100_) canopy coverage for each leaf type, with horizontal bars indicating the time interval between these thresholds. Data were derived from drone imagery. **C)** Aerial imagery of experimental plots 45 d after planting, showing 76-cm spacing (upper panel) and 38-cm spacing (lower panel). The image was captured when 38-cm plots approached 100% canopy coverage while 76-cm plots reached approximately 60% coverage. **D)** Three-dimensional point cloud visualizations (visualized in 2 dimensions) from representative broad-leaf (upper panel) and narrow-leaf (lower panel) plots at Energy Farm. Point clouds are displayed in green against a dark blue background. Peak LAI values, peak biomass values, leaf shape classification, and number of point clouds (*n*) are indicated for each plot. **E)** Time course of digital biomass accumulation at Energy Farm under 76-cm spacing. Box and whisker plots show distributions at 14 time points, with the horizontal rectangle indicating vegetative and reproductive growth stages. **F)** Peak biomass comparison between broad (blue) and narrow (orange) leaf shapes across both spacings. Bars with white stripes represent 38-cm spacing, while solid bars represent 76-cm spacing. Error bars indicate standard deviation. Sample size (*n*) and ANOVA *P*-values are provided. Digital biomass data were collected using LiDAR attached to cable-mounted Spidercam platform. In boxplots: center line, median; box limits, 25th to 75th percentiles; whiskers, 1.5 × interquartile range; points, outliers.

Despite significant differences in LAI, broad and narrow-leaf types achieved similar canopy closure rates, suggesting compensatory mechanisms in canopy development. We analyzed temporal dynamics of canopy coverage (CC) using 20 aerial imaging time points spanning from early vegetative development (17 d after planting) to complete canopy closure at the SoyFACE location (Fig. 5A and B; Figure S6).

Row spacing was the dominant factor controlling the time to canopy closure, with large significant effects on both days to 50% canopy coverage (CC_50_; effect size = 1.81, *P* < 0.0001) and days to complete canopy closure (CC_100_; effect size = 2.0, *P* < 0.0001; Fig. 1B and C). Plots with 38-cm spacing reached CC_50_ and CC_100_ substantially earlier than those with 76-cm spacing (CC_50_: 26 vs. 38 d, CC_100_: 44 vs. 65 d after planting; Fig. 5A to C). The period between half and complete canopy closure was also shorter for 38-cm rows (18 d) compared to 76-cm rows (27 d).

In contrast to the strong effect of row spacing on canopy closure, leaf shape had statistically significant but small effects (CC_50_: effect size = 0.17, *P* = 0.01; CC_100_: effect size = 0.09, *P* = 0.007; Fig. 1B and C). The practical differences between leaf types were minimal, with narrow-leaf lines reaching CC_50_ and CC_100_ only 0.04 to 1.46 d later than broad-leaf lines (*P* < 0.001). This negligible delay in canopy closure occurred despite the substantial reduction in LAI observed in narrow-leaf lines.

### Biomass accumulation responds strongly to row spacing with small effects of leaf morphology

We assessed canopy-level biomass dynamics to determine whether the LAI differences between leaf types translated into significant changes in biomass accumulation. A proxy for digital biomass (BM, m^3^ m^−2^) measurements, which quantify 3-dimensional canopy volume, was recorded at 14 time points throughout the growing season at the Energy Farm location (Fig. 5D to F; Figure S5c).

Row spacing was the predominant factor affecting peak biomass accumulation, with an effect size 4 times larger than that of leaf shape (0.96 vs. 0.24, *P* < 0.0001 and *P* = 0.16, respectively; Fig. 1B and C). Plots with 38-cm spacing achieved 10% higher peak biomass compared to 76-cm spacing (6.11 vs. 5.56 m^3^ m^−2^, *P* < 0.0001; Fig. 5F), consistent with greater LAI.

Leaf morphology had a smaller but detectable influence on biomass accumulation. Broad-leaf lines produced approximately 3% higher peak BM compared to narrow-leaf lines (*P* = 0.009; Fig. 5F), with this difference ranging from 2% in 38-cm spacing to 3.5% in 76-cm spacing. This modest biomass reduction in narrow-leaf lines was proportionally smaller than the 13.3% LAI reduction (reported in Section 3), suggesting compensatory mechanisms in canopy architecture that partially mitigate the impact of reduced leaf area on overall biomass.

We also analyzed peak Normalized Difference Vegetation Index (NDVI) values from drone imagery at SoyFACE, which provides an integrated measure of canopy greenness and density. Interestingly, neither leaf shape nor spacing significantly affected peak NDVI (effect sizes = 0.1 and 0.96, *P* = 0.54 and *P* = 0.70, respectively; Fig. 1B and C). Both spacing treatments achieved near identical peak NDVI values (0.938 vs. 0.936 for 38-cm and 76-cm, respectively, *P* < 0.001), as did both leaf types (0.938 vs. 0.936 for broad and narrow, respectively, *P* < 0.001; Figure S5d and e). This negligible difference (0.25%) in peak NDVI despite measurable differences in LAI and biomass suggests that NDVI saturates at high canopy density, limiting its sensitivity for detecting subtle differences in fully developed canopies.

### Lines with narrow-leaf morphology have altered seed number per pod and maintain grain yield

Despite significant differences in LAI and modest differences in biomass accumulation, narrow-leaf lines maintained yield parity with broad-leaf lines while showing distinctive changes in seed packaging and quality parameters.

**Figure 6.**
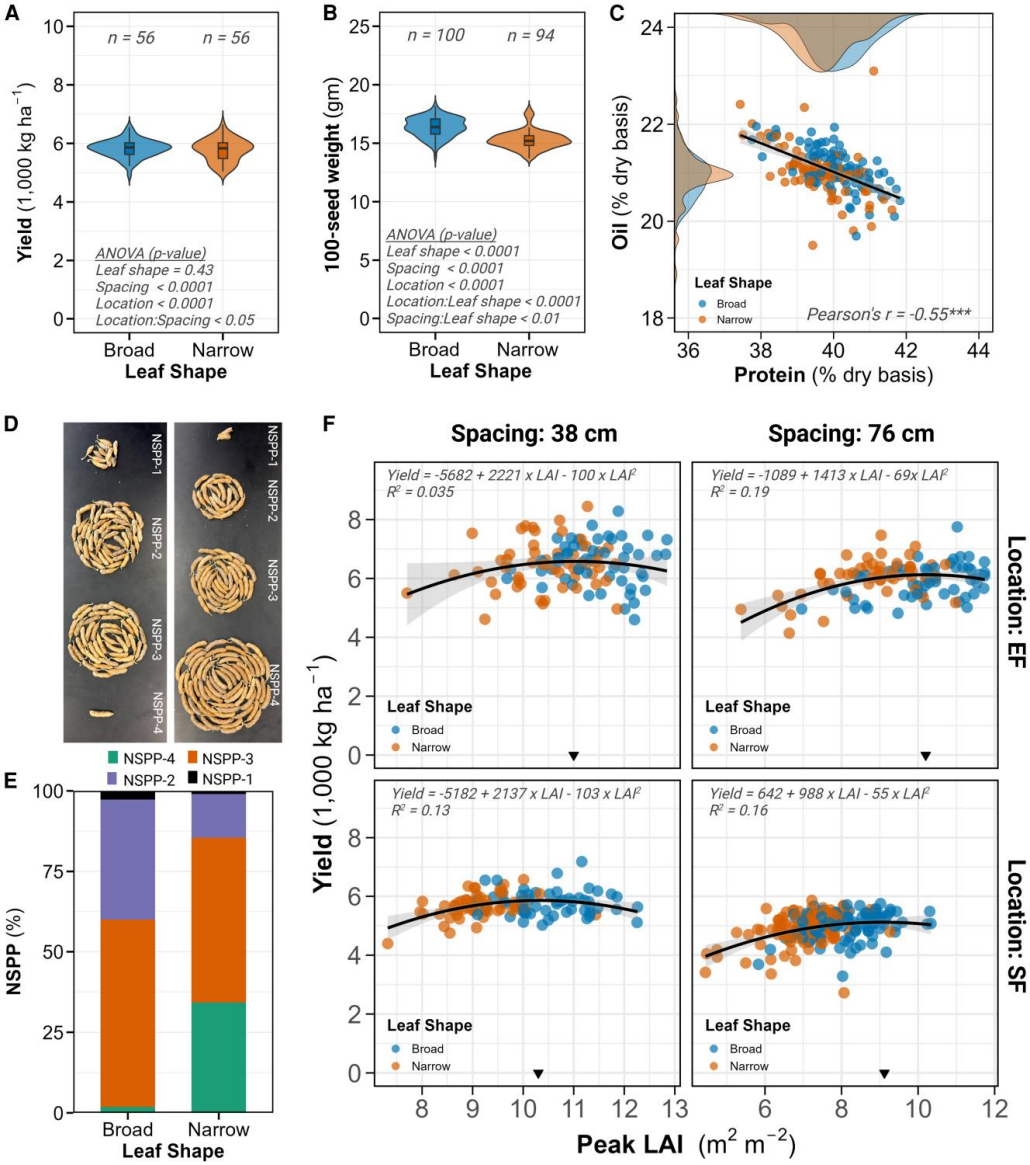
Yield components, seed quality, and yield relationship with LAI. **A)** Violin plot comparing grain yield between broad and narrow-leaf shapes, with sample size (*n*) and ANOVA *P*-values indicated. Values represent BLUEs from the statistical model. **B)** Violin plot comparing 100-SW between broad- and narrow-leaf shapes. Sample size (*n*) and ANOVA *P*-values are provided. Values represent BLUEs from isogenic lines across all locations and spacings. **C)** Scatter plot showing the relationship between seed protein (*x* axis) and seed oil content (*y* axis), with data points colored by leaf shape. The black regression line includes a shaded 95% confidence interval. Density plots for oil and protein content are displayed along the corresponding axes, colored by leaf shape. Pearson correlation coefficient (*r*) is provided with significance indicated by asterisks (*P < 0.05, **P < 0.01, ***P < 0.001). Data include all locations and spacings. **D)** Representative images of soybean pods categorized by seed number per pod for broad-leaf (left panel) and narrow-leaf (right panel) shapes. **E)** Stacked bar chart showing the percentage distribution of seeds per pod (NSPP) for broad and narrow-leaf shapes. Each bar is subdivided to represent the proportion of 4-seeded (NSPP-4, green), 3-seeded (NSPP-3, orange), 2-seeded (NSPP-2, purple), and 1-seeded (NSPP-1, black) pods. Data collected only at SoyFACE 76-cm row spacing. **F**) Quadratic relationship between peak LAI (*x* axis) and grain yield (y axis) for each location and spacing combination. Data points are colored by leaf shape, with black triangles indicating the optimal peak LAI value that maximizes yield. Black curves represent quadratic regression fits with shaded confidence intervals. The quadratic equations and coefficient of determination (*R*^2^) for each model are provided. In boxplots: center line, median; box limits, 25th to 75th percentiles; whiskers, 1.5 × interquartile range; points, outliers.

Location and row spacing strongly influenced grain yield (effect sizes = 0.75 and 0.91, respectively, *P* < 0.0001; Fig. 1B and C), whereas leaf shape had no significant effect (effect size = 0.08, *P* = 0.54). Plots at the Energy Farm yielded 17% greater than plots at SoyFACE (6255.9 vs. 5,331.6 kg ha^−1^, *P* < 0.0001), and 38-cm spacing produced 12% greater yield than 76-cm spacing (6,108.0 vs. 5,449.4 kg ha^−1^, *P* < 0.0001). Most importantly, the yield difference between broad and narrow-leaf isogenic lines was statistically nonsignificant and agronomically negligible (5,801.2 vs. 5,756.3 kg ha^−1^, a difference of only 0.78%, *P* = 0.43; Fig. 6A; Figure S8a). This yield parity was consistent across both locations and spacings treatments, demonstrating that narrow-leaf lines can maintain productivity despite reduced leaf area and biomass.

While yield was unchanged, 100-seed weight (SW) was significantly affected by all 3 factors, with leaf shape exerting the largest effect (effect size = 1.43, *P* < 0.0001; Fig. 1B and C). Seeds from broad-leaf lines were 9% heavier than those from narrow-leaf lines (16.49 vs. 15.01 g per 100 seeds, *P* < 0.0001; Fig. 6B). Location and spacing also influenced SW, with Energy Farm producing 5% heavier seeds than SoyFACE (16.2 vs. 15.4 g, *P* < 0.0001) and 38-cm spacing yielding 2% heavier seeds than 76-cm spacing (15.96 vs. 15.64 g, *P* < 0.0001).

Seed composition analysis revealed small but significant differences in protein content, with leaf shape showing a 4-fold larger effect (effect size = 0.62, *P* < 0.001) than location or spacing (effect sizes = 0.16 and 0.19, *P* = 0.29 and *P* = 0.20, respectively; Fig. 1B and C). Broad-leaf lines had 1.4% higher protein content than narrow-leaf lines (40.32% vs. 39.76%, *P* < 0.0001). Conversely, oil content showed minimal variation between leaf types (20.99% vs. 20.95%, *P* = 0.66) but varied significantly by location and spacing (effect sizes = 0.85 and 0.73, *P* < 0.0001). The inverse relationship between protein and oil was apparent in the narrow and broad-leaf lines (*r* = −0.55, *P* < 0.0001; Fig. 6C).

The most striking difference between leaf types appeared in seed distribution patterns. Analysis of pod characteristics from SoyFACE plots with 76-cm spacing revealed dramatic differences in the number of seeds per pod (NSPP) despite no significant differences in total pod number per plant (40 vs. 39 for broad vs. narrow, effect size = 0.11, *P* = 0.44; Fig. 1B and C; Figure S8c). Narrow-leaf lines had many 4-seeded pods, with NSPP-4 accounting for 34% of total pods compared to only 1.8% in broad-leaf lines (effect size = 1.72, *P* < 0.0001; Fig. 6D and E). Corresponding reductions occurred in 3-seeded pods (51.2% vs. 58.2%), 2-seeded pods (13.6% vs. 37.3%), and 1-seeded pods (1% vs. 2.7%) in narrow vs. broad-leaf lines (*P* < 0.001). This increase in 4-seeded pods in narrow-leaf lines effectively compensated for their 9% reduction in individual SW, resulting in the observed yield parity between leaf types.

### Canopy optimization for yield follows a nonlinear relationship with peak LAI

To understand the functional relationship between canopy development and productivity, we analyzed the mathematical relationship between peak LAI and grain yield across all treatment combinations. When comparing linear versus quadratic models of the LAI-yield relationship, the quadratic model consistently provided superior fit across all location–spacing combinations. Model comparison metrics strongly favored the quadratic relationship, with lower AIC values (3,593 vs. 3,616), generally lower BIC values (3,637 vs. 3,651), reduced error (RMSE: 31.8 vs. 32.9), and higher variance explanation (*R*^2^: 0.52 vs. 0.28) cumulatively across all conditions (Figure S8b). This indicated that yield does not increase linearly with increasing LAI but rather reaches a maximum at an optimal LAI value before declining.

The estimated optimal LAI values for maximizing yield varied by treatment combination, ranging from 9.06 (SoyFACE, 76-cm spacing) to 11.0 (Energy Farm, 38-cm spacing). The intermediate values were remarkably similar for the remaining conditions: 10.2 for Energy Farm with 76-cm spacing and 10.3 for SoyFACE with 38-cm spacing (Fig. 6F). The moderately low *R*^2^ values of these quadratic models (ranging from 0.035 to 0.19) indicate that while LAI is an important determinant of yield, substantial yield variability is explained by other factors.

### Multivariate analysis of leaf traits and canopy architecture

While the quadratic relationship between LAI and yield provided insight into canopy optimization, it represented only one component of the complex trait network determining soybean productivity. Therefore, to synthesize the complex relationships among the leaf morphological, canopy architecture and yield components traits, 2 complementary multivariate approaches were employed: principal component analysis (PCA) and random forest (RF) regression. PERMANOVA analysis of the multivariate data structure revealed that while leaf shape, spacing, and location had statistically significant effects on trait relationships, the interaction effects explained minimal variation (<0.4%), supporting a global approach to multivariate analysis rather than treatment-specific analyses (Figure S9).

#### PCA reveals coordinated variation in leaf and canopy traits

**Figure 7.**
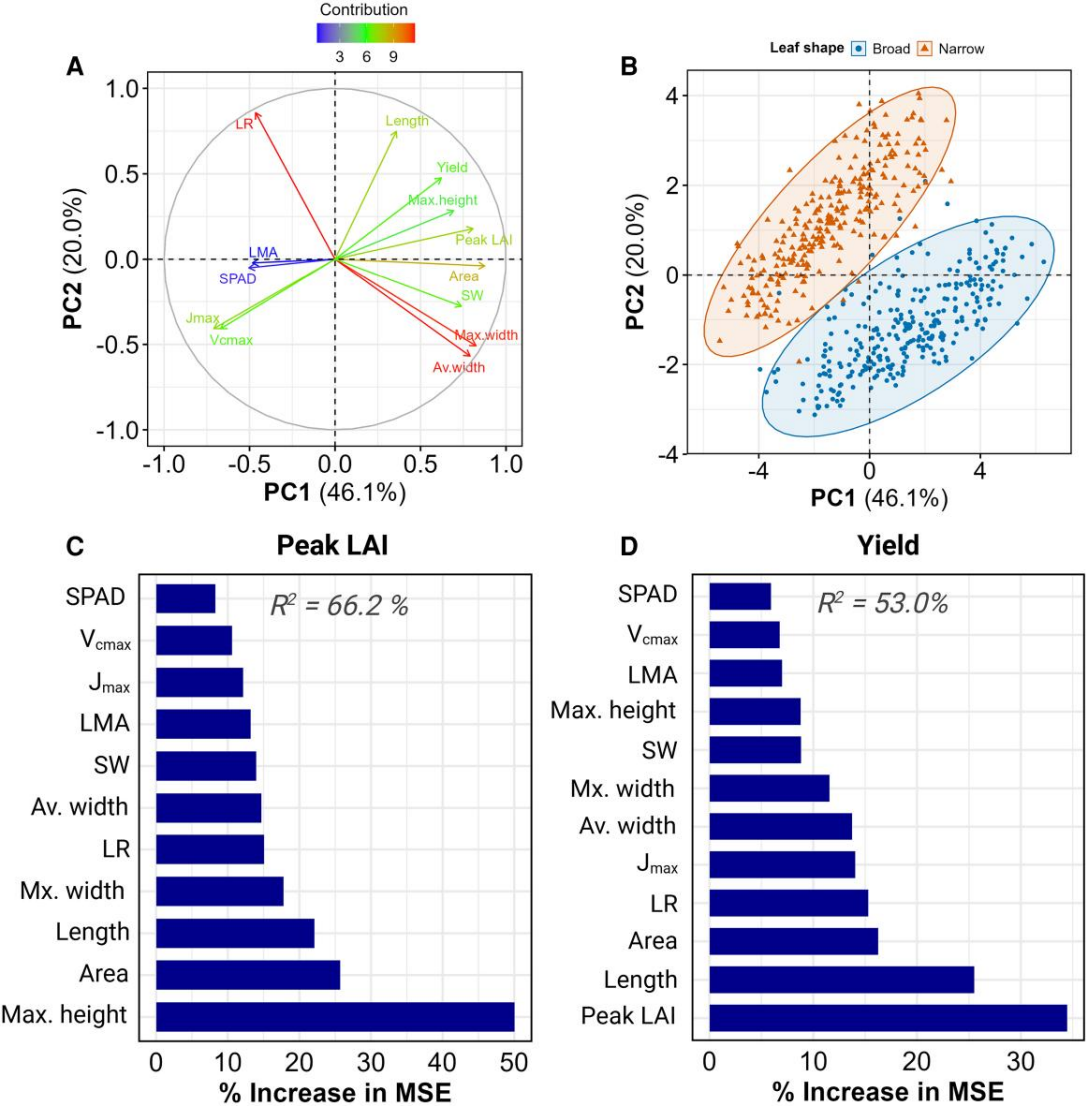
Multivariate analysis of leaf shape effects on yield and related traits. **A)** PCA loading plot displaying the contribution and directionality of individual variables to PC1 and PC2. Arrows represent variables colored according to their contribution magnitude, ranging from highest (red) to lowest (blue) with intermediate values in green. **B)** PCA of phenotypic traits showing PC1 and PC2, which collectively explain the primary variance in the dataset. Individual data points represent lines from all locations and spacings, colored by leaf shape (broad: blue circles, narrow: orange triangles). Two larger central points indicate group centroids, with shaded ellipses demarcating the distribution of each leaf shape category. **C** and **D)** RF variable importance plots for peak LAI **C)** and yield **D)**. The *x* axis displays the percent increase in mean square error (MSE) when each predictor variable (*y* axis) is randomly permuted, indicating the variable's predictive importance. Model fit (*R*^2^) values are provided with each panel.

PCA identified 2 primary axes of variation that together explained 66.1% of the total variance in trait relationships. The first principal component (PC1), accounting for 46.1% of the variance, was strongly associated with leaf morphological and LAI traits (loadings > 0.8) (Fig. 7A). Notably, photosynthetic traits (V_cmax_ and J_max_) and LMA were negatively correlated with PC1, demonstrating a physiological trade-off with leaf structural traits. PC2 explained an additional 20% of the variance and was primarily defined by LR (loading = 0.85) and leaf length (loading = 0.77), suggesting a separate axis of variation for leaf shape independent of size. When individual lines were plotted along these components, broad-leaved and narrow-leaved isogenic lines separated primarily along PC2, with minimal overlap between groups (Fig. 7B). This separation confirms that our introduced genetic variation primarily affected leaf shape.

#### RF analysis ranks the relative importance of traits for predicting yield and peak LAI

RF regression models were constructed to identify key predictors of peak LAI and grain yield, with the models explaining 66.2% and 53.0% of the variance, respectively. For peak LAI, plant height was the strongest predictor (50.0% increase in MSE), followed by leaf area (25.7%) and leaf length (22.1%) (Fig. 7C). Interestingly, leaf ratio ranked relatively lower in importance for peak LAI prediction (15.1%), although leaf ratio is correlated with both leaf area and leaf length. For grain yield prediction, peak LAI emerged as the most important predictor variable, with a 34.5% increase in MSE, followed by leaf length (25.5%), leaf area (16.2%), and leaf ratio (15.3%) (Fig. 7D). This hierarchy underscores the primary importance of canopy development for determining yield potential, with leaf morphological traits playing significant but secondary roles.

## Discussion

Agricultural productivity hinges on balancing resource investment. The Green Revolution of the 1960s significantly boosted harvest indices in crops through optimized resource use (Miflin 2000). However, modern crops face new challenges in resource allocation. Artificial selection in modern varieties has indirectly led to maximizing leaf surface area (reviewed in Slattery and Ort 2021). Combined with rising atmospheric CO_2_ concentrations, this trend can result in overinvestment in leaf development, diverting resources from reproductive growth to vegetative structures (Ainsworth and Long 2005; Srinivasan et al. 2017; Soares et al. 2021). To address this, we developed isogenic soybean lines with reduced leaf area by introducing narrow-leaf morphology into a broad-leaf elite modern variety. Evaluating these lines across 2 distinct environments and agronomically relevant row spacings enabled us to assess how reduced leaf investment influences canopy structure, physiology, and yield outcomes.

### Leaf shape optimization balances resource allocation

Narrow-leaf morphology in soybeans improved resource allocation, maintaining yield despite a 13% decrease in LAI and 3% reduction in biomass (Figs. 2C, 5F, and 6A). Narrow-leaf shape did not change yield (<1% difference, *P* = 0.43) across environments and row spacings. These results align with prior studies that compared productivity of narrow and broad-leaf soybeans (Mandl and Buss 1981; Wells et al. 1993; Dinkins et al. 2002). In contrast, Cai et al. (2021) reported a ∼9% yield increase in narrow-leaf mutants of a low-latitude cultivar in China while Bianchi et al. (2020) found yield benefit only under high-density planting in Argentina, reflecting important genotype × environment interactions. Our study of over 200 BC_3_F_2:4_ lines suggest that reducing vegetative investment may improve the harvest index and promote yield efficiency in soybeans. The maintenance of yield despite reduced leaf area implies that narrow-leaf lines achieve greater return on structural investment potentially through enhanced light penetration to lower canopy layers, reduced self-shading, and enhanced photosynthesis (Bianchi et al. 2020).

The 5% increase in LMA observed in narrow-leaf lines likely results from modified tissue organization rather than enhanced photosynthetic machinery. Our previous anatomical work demonstrated that natural variants of narrow leaves are approximately 30 µm thicker for every unit increase in leaf ratio, with this increased thickness containing more mesophyll cell layers per unit area (Tamang et al. 2023). This resulted in higher (∼23%) area-based photosynthetic rates (*A*) despite similar per-cell photosynthetic capacity. While we did not directly measure *A* in the present study, the similar structural modifications observed in our NILs suggest this mechanism is likely to operate here as well. The lack of proportional increase in V_cmax_ despite higher LMA, coupled with only modest increase in J_max_ (∼4%) (Fig. 3D), confirms that any photosynthetic enhancement stems from having more photosynthetic tissue packed into each unit area rather than improved biochemical capacity per se. This is consistent with Poorter et al. (2009) who showed that LMA increases from structural tissues alone do not necessarily enhance photosynthetic capacity per unit mass but can affect area-based performance. Our soybean study parallels findings in rice, where NAL1 mutations increased mesophyll cell density and leaf thickness, leading to enhanced photosynthetic rates through increased photosynthetic tissue per area (Takai et al. 2013). The correlation between LMA and photosynthetic parameters observed across our population (Fig. 3E) reflects the complex genetic variation influencing both leaf structure and function. This pattern aligns with the optimal resource allocation theory (Bloom et al. 1985), which posits that plants distribute resources to optimize fitness under constraints. In agricultural contexts, where reproductive output (yield) is the primary fitness metric, the narrow-leaf trait may help optimize the trade-off between vegetative and reproductive investment.

### Narrow-leaf morphology maintains canopy coverage likely through architectural compensation

Early canopy coverage is critical in agricultural crops, as it enhances light interception, suppresses weed growth, and maximizes photosynthetic productivity during key early vegetative growth stages (Richards 2000; Purcell et al. 2002). Despite a 13% reduction in peak LAI (Fig. 4C), our narrow-leaf isogenic soybean lines showed no difference in canopy development dynamics. The negligible delay in canopy closure (less than 2 d delay to achieve 100% canopy closure; Fig. 5A and B) and only modest reductions in biomass accumulation (3% lower at high yielding location Energy Farm, Fig. 5F) suggest architectural adaptations that partially mitigate the impact of reduced leaf area. This compensation likely involves alterations in leaf arrangement, canopy structure, and light interception patterns that collectively maintain effective resource capture despite the modified leaf morphology (Niinemets 2010; Sreekanta et al. 2024). While LAI and biomass differed significantly, NDVI did not vary between leaf types or spacings at peak canopy size (Figure S6d and e). This saturation of NDVI under high-density canopies emphasizes the limitations of traditional greenness indices for distinguishing subtle morphological effects and suggests the need for integrating structural or multispectral indices in high-resolution phenotyping.

### Nonlinear relationship between LAI and yield

We observed a nonlinear (quadratic) relationship between peak LAI and grain yield, where yield peaked at an optimal LAI and declined thereafter (Fig. 6F). This optimal LAI varied with leaf type, location, and spacing, ranging from 9 (SoyFACE, 76-cm spacing, narrow leaves) to 11 (Energy Farm, 38-cm spacing, broad leaves). These values exceed earlier thresholds (eg., LAI = 7.5 in Srinivasan et al. 2017), likely due to differences in genotype, planting density, management, and environmental conditions.

This nonlinear relationship between canopy size and seed yield supports the hypothesis that LAI in soybean is too high (Srinivasan et al. 2017). Beyond the optimal LAI, excess foliage may increase respiration, mutual shading or create microclimate stress, reducing reproductive output (Board and Harville 1992; Setiyono et al. 2008; Tagliapietra et al. 2018; Digrado et al. 2020). Narrow-leaf lines may inherently position plants closer to this optimum, achieving high yield with reduced vegetative cost. Supporting this, our RF regression identified peak LAI as the most important trait predicting grain yield, followed by leaf architectural traits (Fig. 7D). These results suggest that optimizing whole-plant architecture has a stronger influence on yield than leaf shape alone, highlighting the importance of integrated phenotypic strategies in soybean ideotype design.

### Environmental context dependency of leaf shape effects

The adaptive value of architectural traits, including leaf morphology, is highly contingent on environmental conditions (Sarlikioti et al. 2011). Krause et al. (2025) highlighted the importance of environmental context by defining soybean mega-environments. Our study underscores this, with location significantly influencing traits such as V_cmax_, J_max_, peak LAI, and yield (Figs. 1B and 3C and D). Energy Farm, the higher productivity site, had 17% higher grain yield, 16% higher peak LAI, and 5% heavier seeds than SoyFACE (Figs. 4C and 6A and B).

Interestingly, V_cmax_ and J_max_ were significantly higher at SoyFACE (17% and 12%, respectively, Fig. 3C and D), indicating that in resource-limited environments, soybeans increase photosynthetic capacity per unit leaf area to compensate for lower canopy development. We suspect that greater fertility at the Energy Farm enabled greater total leaf area (∼44% more), allowing nitrogen to be distributed across more tissue. This led to slightly lower nitrogen concentration per leaf, evidenced by 3% lower SPAD readings, and correspondingly lower photosynthetic capacity (Evans 1989; Poorter and Evans 1998; Walker et al. 2014). These results support the hypothesis that elevated photosynthetic capacity is particularly important in stressful environments, a key insight for breeding resilient soybean varieties (Flood et al. 2011).

### Row spacing effects on canopy architecture and yield

Row spacing strongly influences resource competition by altering plant spatial distribution (Board et al. 1990; Andrade et al. 2002). Narrower rows promote faster canopy closure and greater light capture and suppress weed density, biomass, and weed seed production (Singh et al. 2023). In our study, canopy closure was 3 wk earlier in narrow rows, associated with a 20% increase in LAI (Fig. 4C), 10% increase in biomass (Fig. 5F), and 12% yield boost (Fig. 6A). These benefits were consistent across leaf types, suggesting that optimized morphology and management practices can be combined to enhance resource use efficiency. This supports broader findings by Andrade et al. (2019), who documented up to 18% yield increases in narrow rows across nearly 5,000 US soybean fields, especially under late planting or short-season conditions. A trade-off between narrow row spacing and disease pressure has also been reported (Webster et al. 2022), so the economic gains of greater yields in narrow row spacing would need to be considered against greater costs of fungicides by growers.

### Ecological context and breeding implications

Leaf morphology represents a fundamental axis of plant functional diversity that influences light capture, water relations, gas exchange, and herbivore defense across ecosystems. Our findings partially align with leaf economic spectrum (LES; Wright et al. 2004) theory—narrow-leaf lines invest in higher LMA with reduced total leaf area, signaling conservative resource use strategies. However, they deviate by maintaining yield despite this reduced leaf area, primarily through increased reproductive efficiency. Such within-species variations often challenge global LES patterns, revealing more complex resource allocation strategies than simple trade-offs, particularly in crop species where breeding has selected for novel trait combinations that optimize productivity (John and Garnica-Diaz 2023).

These results emphasize the need to reassess traditional breeding priorities. Historically, selection for high yield has resulted in broad leaves and high LAI to maximize early-season growth and light interception. However, our study shows that narrow-leaf traits can reduce vegetative costs without sacrificing yield, potentially contributing to greater efficiency under both high-input and stress-prone environments. Given increasing climate variability and input limitations, this shift in breeding priorities could enhance crop resilience.

The narrow-leaf phenotype is largely controlled by the *ln* locus encoding a JAGGED-like transcription factor *GmJAG1* (Jeong et al. 2012), which simplifies introgression into elite backgrounds. Previous work has demonstrated that despite *GmJAG1*'s pleiotropic effects on leaf shape and seed number per pod, there are no negative impacts on key agronomic traits including yield, maturity or photosynthetic capacity (Dinkins et al. 2002; Sayama et al. 2017; Cai et al. 2021), which supports its utility in breeding pipelines. This is further reinforced by our PCA showing clear separation of leaf types while other traits remained largely unchanged (Fig. 7B). This validates the value of these near-isogenic lines for physiological studies.

The near-isogenic lines developed here, which differ mainly in leaf morphology, provide valuable materials for physiological modeling. They offer unique opportunities to isolate the effects of LAI and canopy structure on productivity in a controlled genetic background, enabling more precise parameterization of crop growth models and supporting efforts to simulate genotype × environment × management interactions.

### Future directions

Our field study was done in East Central Illinois, in the heart of the US Corn Belt. Future research should evaluate the performance of narrow-leaf genotypes in a broader spectrum of environmental conditions, including regions prone to water limitation, high temperatures, or suboptimal planting densities. Such conditions may accentuate the advantages of reduced leaf area, improving water use efficiency and heat dissipation while minimizing excessive vegetative growth. In addition, mechanistic studies on carbon partitioning and source-sink dynamics in narrow-leaf lines would provide insights into how these genotypes maintain yield. Investigating the interaction between leaf shape and other architectural traits—such as leaf angle, plant height, branching architecture, and rooting depth—could help refine ideotype models tailored to different agroecological zones.

From a technological perspective, integrating narrow-leaf morphology with novel plant protection strategies could improve performance in places with high disease load. Additionally, the narrow-leaf trait could be combined with strategies to enhance water use efficiency, abiotic stress resistance, photosynthesis, sink strength, or nutrient uptake to enhance performance in suboptimal environments (Bailey-Serres et al. 2019). Fast-tracking combinations of strategic traits could enhance the development of climate-resilient, resource-efficient soybean varieties tailored for sustainable production in a changing world.

## Conclusion

Based on the analysis of 204 isogenic soybean lines, this study demonstrates that narrow-leaf morphology is a promising strategy for optimizing resource allocation without compromising grain yield. Despite reducing LAI by 13% and biomass by 3%, narrow-leaf lines maintained yield parity with broad-leaf counterparts across different environmental conditions and management practices, achieving greater return on structural investment. The narrow-leaf lines had similar canopy closure dynamics and photosynthetic capacity, but altered seed packaging patterns with significantly more 4-seeded pods. The nonlinear relationship between peak LAI and yield supports the hypothesis that modern soybean varieties may overinvest in vegetative structures at the expense of reproductive efficiency. These findings challenge traditional breeding paradigms that have resulted in high leaf area and suggest that the single-gene control of narrow-leaf morphology through *GmJAG1* offers a tractable approach for developing resource use efficient cultivars that maintain productivity while reducing metabolic costs associated with excessive canopy development. We offer a strategy to test for increasing sustainable agriculture under intensifying climate variability and resource constraints.

## Materials and methods

### Parent selection and development of isogenic lines via repeated backcrossing

To develop isogenic soybean lines that varied for leaf morphology, we selected the broad-leaved cultivar LD11-2170 (Maturity Group III, MG III) as the recurrent parent. Developed at the University of Illinois, LD11-2170 has served as a reference check in the Uniform Soybean Tests of the Northern Region since 2019, owing to its consistent high field trial yields (>4,700 kg per hectare) and favorable agronomic traits (Cai and Brock 2023). For the narrow-leaf trait, 2 donor lines PI 612713A (MG I) and PI 547745 (MG II) were identified from a 2020 leaf shape survey of the USDA germplasm collection conducted in Urbana, Illinois (Tamang et al. 2023). PI 612713A originated from Heilongjiang Sheng, China, and was deposited in USDA National Plant Germplasm System (NPGS) in 1999. Similarly, PI 547745 was developed at IL and deposited in NPGS in 1975 (npgsweb.ars-grin.gov). These recurrent and donor lines were further characterized in summer 2022, during which the known leaf shape gene *GmJAG1* (Jeong et al. 2012) was sequenced to confirm the presence or absence of its allele.

Initial crosses to produce F_1_ hybrids were performed in summer 2021 at the University of Illinois. Three successive backcrosses were subsequently conducted to introgress the narrow-leaf trait into the LD11-2170 background from each donor line. During the backcrossing, Kompetitive Allele-Specific PCR (KASP) markers developed from the *GmJAG1* gene were used to select F_1_ plants that were heterozygous for this gene. From the resulting BC_3_F_1_ generation, 600 seeds derived equally from the 2 narrow-leaved donors were planted. Segregating BC_3_F_2:3_ lines were screened with the KASP marker, yielding 209 homozygous lines: 106 broad-leaved and 103 narrow-leaved (116 from the PI 547745 parent and 93 from the PI 612713A parent).

The BC_3_F_2_ plants were grown and 204 BC_3_F_2:3_ lines were advanced for seed increase. Seeds from these lines (BC_3_F_2:4_) were subsequently planted in experimental plots for this study. The 3 parental lines (LD11-2170, PI 612713A, and PI 547745) and the 204 BC_3_-derived lines were genotyped using the Agriplex 1K Soy SNP panel, comprising 1,327 SNPs (Agriplex Genomics, Cleveland, Ohio, United States).

### Field experimental design, planting, and management practices

The experimental plots were arranged in a Type II Modified Augmented Design (MAD) (Lin and Poushinsky 1985). This implementation of the Type II MAD specifically addresses the rectangular nature of soybean field plots by arranging plots in rows within blocks. As described by Lin and Poushinsky (1985), this arrangement ensures optimal spatial control while accommodating row-based planting systems. Each block contained 25 plots with the primary check (LD11-2170) positioned centrally to maintain equal distances to surrounding test plots, maximizing the effectiveness of spatial adjustment. Secondary checks (PI 612713A and PI 547745) were randomly distributed within blocks to capture additional spatial variation that might not be accounted for by the primary check alone.

Field experiments were conducted at 2 experiment farms, SoyFACE (SF) and Energy Farm (EF) in 2 row spacings (38 cm and 76 cm), at the University of Illinois, Urbana-Champaign, United States, approximately 2 miles apart. Of the 204 BC_3_F_2:4_ lines, a set of 112 (56 broad-leaved and 56 narrow-leaved isogenic lines) were planted at both locations and spacings. The remaining second set of 92 BC_3_F_2:4_ lines were planted at SoyFACE with a 76-cm spacing. At SoyFACE, this resulted in 9 blocks for 76-cm spacing and 5-blocks for 38-cm spacing. At Energy Farm, 10 blocks were established for each spacing. Each block contained 25 plots, with isogenic lines randomly assigned to achieve approximately equal numbers of broad- and narrow-leaved lines. Across both locations and spacings, a total of 600 plots were planted. Each plot consisted of four 2.4 m rows, separated by 0.9 m alleys. At both locations, the 2 experimental plots (plots planted at 38-cm and 76-cm row spacing) were surrounded by 4 border rows of a commercial soybean variety on all sides. Seeds were mechanically planted at 314,000 seeds per hectare for 76-cm spacing and 447,000 seeds per hectare for 38-cm spacing. Planting occurred on 30 May 2024 at Energy Farm and 4 June 2024 at SoyFACE.

Weeds were manually removed as needed. Irrigation was applied once at SoyFACE and twice at Energy Farm during the early vegetative growth stages.

### Data collection

Growth stages were determined following the standard soybean developmental stages described by Fehr et al. (1971).

### Leaf morphology and LAI

Leaf morphology was characterized by nondestructively measuring total leaf area, length along the midrib, and widths (average and maximum) using a portable leaf area meter (LI-3000C; LI-COR Biosciences, Lincoln, Nebraska, United States). These measurements were taken at the V5 to V7 growth stage when plants had 5 to 7 fully developed trifoliate leaves. Leaf ratio (LR), defined as the ratio of length to maximum width (Chen and Nelson 2004), was calculated from these measurements. Data were collected once from the middle trifoliate, fully expanded leaf between the 5th and 7th nodes. Leaves destructively harvested for additional measurements (eg, A-Ci response curves, see below) were also scanned with the leaf area meter to create paired datasets with those variables, as leaf ratios varied slightly by node. Relative greenness of all samples leaves was assessed using a SPAD meter (SPAD-502; Konica Minolta, Tokyo, Japan). These leaves were then dried at 65 °C for 1 wk, and their dry mass was recorded to calculate leaf mass per area (LMA).

LAI was measured throughout the season using the SS1 Canopy Analysis System (Delta-T Devices, Cambridge, United Kingdom), a 1-m probe equipped with 64 photosynthetically active radiation (PAR) sensors. The probe was placed perpendicular to the 4 rows of each plot at the plant base. Ten LAI measurements were taken per plot across all 600 plots at both locations (SoyFACE and Energy Farm), totaling 6,000 measurements, from early vegetative to late reproductive stages (25 to 95 d after planting corresponding to V2 through R6 growth stages).

### A-C_i_ response curve measurements

Photosynthetic efficiency of the isogenic lines was evaluated by measuring photosynthetic rates (A) in response to varying intercellular CO_2_ concentrations (C_i_), following established protocols (Long and Bernacchi 2003; Busch et al. 2024). Measurements were conducted using a portable infrared gas exchange analyzer (LI-6800; LI-COR Biosciences, Lincoln, Nebraska, United States) on leaf samples from 264 plots (135 from SoyFACE, 129 from Energy Farm), representing 127 broad-leaved and 137 narrow-leaved lines. Samples were collected at the R1 to R2 developmental stage, except for the early-maturing narrow-leaved donor parents (PI 612713A and PI 547745), which were at R3. An undamaged, unshaded, recently matured trifoliate leaf (typically the fourth from the top node) from the middle rows of each plot was tagged 1 d prior to measurement. The following predawn, tagged leaves were cut at the petiole base, immediately submerged in water, and stored in a cool, dark cabinet to maintain turgor and prevent photosynthetic initiation. Before measurements, leaves were exposed to low light for at least 30 minutes. The LI-6800 was equipped with a 6 cm^2^ circular cuvette, which was clamped onto the middle section of each leaf to accommodate narrower morphologies. Steady-state photosynthetic assimilation rates were recorded at 14 reference CO_2_ concentrations: 400, 300, 200, 100, 75, 50, 25, 400, 400, 600, 800, 1,000, 1,200 and 1,800 ppm. A light intensity of 2,000 µmol m⁻^2^ s⁻^1^, leaf temperature of 28 °C, relative humidity of 65%, fan speed of 10,000 rpm, and airflow of 700 µmol s⁻^1^ were maintained in the cuvette.

Data were collected over 4 d using 9 infrared gas analyzers, with samples grouped by location and row spacing. At the Energy Farm, leaves from 76-cm row spacing plots (*n* = 62) were measured on 23 July 2024, followed by 38-cm row spacing plots (*n* = 67) on 24 July 2024 (54 to 55 d after planting). At SoyFACE, leaves from 76-cm row spacing plots (*n* = 74) were measured on 29 July 2024 and 38-cm row spacing plots (*n* = 61) on 30 July 2024 (55 to 56 d after planting).

### Canopy coverage, NDVI, and digital biomass

Canopy coverage of the isogenic lines was quantified using aerial images captured by a DJI Mavic 3 Multispectral Enterprise drone (DJI, Shenzhen, China). Images (captured from an altitude of 29 m with front overlap of 80% and side overlap of 90%) were collected twice weekly between 17 and 99 d after planting (V2 through R6 growth stages) (26 flights). Orthomosaics were generated from these images using Pix4D Fields (version 2.8.0; Pix4D 2024). Canopy coverage was calculated from the red (R), green (G), and blue (B) channels in QGIS (version 3.36; QGIS Development Team 2024) through a 3-step process. First, pixels were classified as soil or vegetation using the Excess Green Index (2G−(R + B)), followed by thresholding to distinguish between the 2. Second, a square boundary layer was defined for the middle 2 rows of each plot, and zonal statistics were used to count total pixels representing vegetation or soil within the boundary. Finally, canopy coverage was expressed as the percentage of vegetation pixels.

Normalized difference in vegetation index (NDVI), an indicator of plant health and proxy for vegetation growth and biomass, was calculated from the same images (collected between V2 and R6 growth stages) using the near-infrared (NIR) and red (R) channels with the formula (NIR−R)/(NIR + R) in QGIS (3.36; QGIS Development Team 2024). A boundary box encompassing all 4 rows of each plot was created, and zonal statistics provided mean and median NDVI values per plot. Given the high correlation between mean and median NDVI (*r* = 0.95), mean NDVI was selected for all subsequent analyses. These 2 variables, canopy coverage and NDVI, were captured only at SoyFACE.

Biomass at the canopy scale was nondestructively measured at 14 different time points (between 11 and 84 d after planting between V2 and R5 growth stages) using a LiDAR sensor (OUSTER OS0-128, Ouster Inc., San Francisco, California, United States) mounted on a Spidercam system (Ross Video Limited, Ottawa, Canada) at Energy Farm. The measurement platform was commanded to scan the plots at a constant rate of 450 mm s^−1^ to record a series of LiDAR frames which were algorithmically converted into point clouds. The digital biomass of the plot was determined by computing the plot volume and dividing the volume by the plot area, where the plot volume was computed using voxel grid algorithm from the open-source Point-Cloud Library (Rusu and Cousins 2011).

### Plant height and developmental stages

Plant height was manually measured from the base of the plant to the uppermost leaf at all locations and row spacings at 6 time points, from 25 to 95 d after planting (corresponding to V3, V6, R1, R3, R5, and R7 growth stages). Concurrently, developmental stages were monitored and recorded at 6 time-intervals throughout the growing season. These observations enabled the determination of key phenological parameters, including the duration of the vegetative growth phase, days to initiation of the reproductive stage or beginning of flower bloom (R1), and days to full physiological maturity (R8).

### Yield, yield components, and quality

#### Pod number and number of seeds per pod

Pod number (PN) and number of seeds per pod (NSPP) were assessed exclusively at SoyFACE, within plots established at a 76-cm row spacing. At maturity, a representative plant was selected from each of 190 plots, consisting of 187 isogenic lines and 3 parental genotypes. For each plant, the total number of pods was manually counted. Pods were subsequently categorized and counted according to the number of seeds per pod (NSPP), specifically distinguishing among 1- (NSPP-1), 2- (NSPP-2), 3- (NSPP-3), and 4-seeded (NSPP-4) pods.

#### Grain yield, SW, protein, and oil content

Grain yield was determined at maturity (R8 stage) by mechanically harvesting all the plots at both locations and spacings using an ALMACO R1 Single-Plot Rotary Combine (ALMACO, Nevada, Iowa, United States). Harvest data, including total plot weight and grain moisture content, were recorded using the combine's integrated Weight Hopper System. For plots established with 76-cm spacings, the central 2 rows were harvested, whereas all rows were harvested from plots with 38-cm row spacings. Yield data were standardized to a moisture content of 13%. Additionally, 3 random subsamples of 100 seeds each were collected from every harvested plot and weighed to determine seed sizes.

Seed protein and oil content (% dry weight basis) were determined on the same samples used for SW assessment (R8 stage), using an NIR instrument (DA 7250 NIR analyzer, PerkinElmer, Waltman, Massachusetts, United States). The instrument was calibrated using 10 reference samples with known protein and oil content values previously determined through standard wet chemistry methods. Calibration biases were adjusted based on these reference values, while using the manufacturer's preset calibrations as a baseline.

### Statistical analysis

All data processing, statistical analyses, and visualizations were conducted using R (version 4.2.1, R Core Team 2024). Descriptive statistics were calculated for all variables, including mean, standard deviation and range. Pearson correlation coefficients (*r*) were computed to assess relationships among variables, with significance established at *P* < 0.05. For group comparison, independent sample *t*-tests were employed for 2-group comparisons, while analysis of variance (ANOVA) with post hoc Tukey's Honestly Significant Difference tests were used for multiple group comparisons. Data wrangling was performed using *dplyr* (version 1.1.4; Wickham et al. 2023a) and *tidyr* packages (version 1.3.1; Wickham et al. 2023b), and most of the plots were generated with the *ggplot2* package (version 3.5.1; Wickham 2016).

### Estimating parameters from time course measurements of LAI, CC, NDVI, PH, and BM

For traits measured over multiple time points such as LAI, canopy coverage (CC), normalized difference in vegetation index (NDVI), plant height (PH), and digital biomass (BM), key temporal parameters were extracted by evaluating 3 curve-fitting models (LOWESS; locally weighted scatterplot smoothing, logistic, and quadratic with second-degree polynomial). Model selection employed a comprehensive approach, evaluating both traditional fit quality metrics and information criteria. Traditional metrics included mean absolute error (MAE), residual sum of squares (RSS), root mean squared error (RMSE), and the coefficient of determination (*R*^2^), while information criteria assessment used Akaike information criterion (AIC) and Bayesian information criterion (BIC) to account for model complexity. For all traits, logistic models failed to converge during information criteria assessment indicating these traits did not follow a sigmoidal pattern in our experimental context. Formal statistical testing (ANOVA with Tukey HSD) of the traditional metrics confirmed significant differences between models. Across all traits, the LOWESS model consistently showed superior performance, with lower MAE, RSS, RMSE, AIC, and BIC and higher *R*^2^ values (Figure S10). As a result, the following parameters were extracted from the LOWESS fits: peak LAI, days to 50% canopy coverage (CC_50_), days to 100% canopy coverage (CC_100_), peak digital biomass, peak NDVI, days to start of NDVI decline, and maximum plant height.

### Predicting V_cmax_ and J_max_ from A-C_i_ curves

Photosynthetic parameters, maximum rate of Rubisco activity (V_cmax_) and maximum rate of RuBP regeneration (J_max_), were estimated from A-C_i_ curves using *PhotoGEA* R package (version 1.1.0; Lochocki et al. 2025).

The *PhotoGEA* package implements a whole-curve fitting approach using maximum likelihood regression and derivative-free optimization algorithms. For our analysis, we employed the *fit_c3_aci* function, which fits A-Ci curves using the Farquhar-von-Caemmerer-Berry (FvCB) model for C_3_ plants. Mesophyll conductance was assumed to be infinite, yielding apparent photosynthetic parameters based on intercellular CO_2_ concentration (C_i_). All parameter estimates were normalized to 25 °C using the temperature response functions of Bernacchi et al. (2001).

### Adjustment for spatial variability in trait estimates

To address the inherent field heterogeneity present in the trial and its influence on the estimates of the 25 traits, 2 distinct spatial adjustment approaches relevant to the Type II Modified Augmented Design were compared (Method I and Method III, Lin and Poushinsky 1985). The adjustments were made independently across 2 locations (SoyFACE and Energy Farm) and 2 row spacings (38 cm and 76 cm).

The first method (Method I) corresponds to the “adjustment by design structure” approach, which uses an ANOVA model to account for row and column effects through the analysis of control plots. The model follows as


$$Y_{ijk}^{\mathrm{check}1\;}=\mu +R_{i}+C_{j}+\epsilon_{ijk}$$

where $Y_{ijk}^{\mathrm{check}1\;}$ is the observed trait value for the primary check (check1) in row *i*, column *j*, and block *k*, *µ* is the overall mean of the trait for the primary check (check1) across all blocks, *R_i_* represents the effect of the *i*-th row, *C_j_* represents the effect of the *j*-th column, and *ɛ_ijk_* is the residual error.

Using the row and column effects estimated from this model, the adjusted trait value for each test plot was computed as


$$Y_{ij}^{\mathrm{Adjusted}\;}=Y_{ijk}-R_{i}-C_{j}+2\mu$$

where $Y_{ij}^{\mathrm{Adjusted}\;}$ is the adjusted trait value for the test plot in row *i* and column *j*, *Y_ijk_* is the observed trait value of the test plot, *R_i_* and *C_j_* are the estimated row and column effects, and *μ* is the overall mean of the primary check.

Similarly, the second method (Method III) corresponds to the “adjustment by regression” approach but extends it by incorporating both primary and secondary checks. The model follows as


$$Y_{ij}^{\mathrm{Adjusted}\;}=Y_{ij}\text{–}\beta (\mathrm{Check}1_{ij}-\mathrm{Check}2_{2,3}^{\mathrm{Avg}\;})+\alpha$$

where $Y_{ij}^{\mathrm{Adjusted}\;}$ is the adjusted trait value for the test plot, *Y_ij_* is the observed trait value, Check1*_ij_* is the trait value of the primary check in the corresponding block, $\mathrm{Check}2_{2,3}^{\mathrm{Avg}\;}$ is the average trait value of the 2 secondary checks, *β* is the regression slope relating the secondary checks' average to the primary check, and *α* is the regression intercept.

The decision to apply one of the adjustments was based on the relative efficiency (RE) of each approach, defined as


$$\mathrm{RE}=\frac{IWPError_{Original}}{IWPError_{Adjusted}}$$

where *IWPError_Original_* is the intra-whole plot error without adjustment and *IWPError_Adjusted_* is the intra-whole plot error after applying either method.

The intra-whole plot error was calculated as


$$IWPError=\frac{SSdiff_{1}\;+\;SSdiff_{2}}{2.(n_{1}+n_{2}-2)}$$

where *SSdiff_1_* and *SSdiff_2_* are the sums of squared differences for the 2 secondary checks and *n_1_ and n_2_* are the number of blocks for the secondary checks.

For the adjusted values, a similar calculation was made for adjusted sums of squared differences (*SSadj_1_* and *SSadj_2_*). The adjustment decision followed these criteria:

- If both methods resulted in RE <100, no adjustment was applied as precision gains were insufficient.
- If one method had RE ≥100 and the other had RE <100, the method with RE ≥100 was chosen.
- If both methods had RE ≥100, the method with the higher RE was selected for its greater efficiency in reducing spatial variability.

This comparative strategy was applied individually to each of the 25 traits across the 4 location and spacing combinations (Figure S7). For each trait and combination, RE values were computed to determine whether adjustment was warranted and, if so, which method to use. Adjustments were not universally applied, as their necessity depended on RE exceeding 100 or the highest RE when both methods met this threshold.

### Best linear unbiased estimates analysis

For yield, SW, protein, and oil content measured across both locations and spacings, best linear unbiased estimates (BLUEs) were calculated to obtain representative values for each isogenic line, adjusted for location and spacing effects. BLUEs were generated using linear mixed-effects models implemented in the *lme4* R package (version 1.1.36; Bates et al. 2015), with *emmeans* R package (version 1.10.7; Lenth 2025) to extract the marginal means (BLUEs) for each line with the following model structure:


$$Y_{ijk}=Line_{i}+Location_{j}+Spacing_{k}+\varepsilon_{ijk}$$

where $Y_{ijk}$ is the observed trait (grain yield, SW, protein or oil content) value, *Line_i_* is the fixed effect of the *i*^th^ isogenic line, *Location_j_* is the random effect of the *j*^th^ location, *Spacing_k_* is the random effect of the *k*^th^ spacing, and *ε_ijk_* is the residual error.

### Peak LAI–yield relationship analysis

To investigate the relationship between peak LAI and yield, linear and quadratic regression models were fitted to data for each location (SF, EF) and row spacings (38 cm, 76 cm). Model performance was evaluated using AIC, BIC, *R*^2^, and RMSE to identify the best-fitting model for each group.

### Hierarchical mixed-effects modeling framework to assess trait variation

To evaluate how location, spacing, leaf shape, and donor parent affected 25 measured traits, we used hierarchical mixed-effects models implemented in the *lme4* R package (version 1.1.36; Bates et al. 2015). Statistical significance was assessed using the *lmerTest* package (version 3.1.3; Kuznetsova et al. 2017). The preliminary model included 4 fixed factors: donor parent (PI 612713A, PI 547745), location (SoyFACE, Energy Farm), spacing (38 cm, 76 cm), and leaf shape (broad, narrow), along with their interactions and a random intercept for the interaction of leaf shape and isogenic lines. Since the statistical analysis revealed that donor parent did not significantly influence the measured traits (*P* > 0.05), it was removed from the final model:


$$\begin{array}{rl} Y_{ijkl} & =\mu +L_{i}+S_{j}+LS_{k}+(L\times S{)}_{ij}+(L\times LS{)}_{ik}\\ & \;+(S\times LS{)}_{\;jk}+(L\times S\times LS{)}_{ijk}+b_{kl}+\varepsilon_{ijkl} \end{array}$$

where $Y_{ijkl}$ is the standardized trait value (z-score), *μ* is the overall mean, *L_i_* is the fixed effect of location (SoyFACE, Energy Farm), *S_j_* is the fixed effect of spacing (38 cm, 76 cm), and *LS_k_* is the fixed effect of leaf shape (broad, narrow), All interaction terms are denoted by products (eg, (*(L*  *×*  *S)_ij_*), *b_kl_* is the random effect for the interaction of leaf shape and isogenic line *l*, with *b_kl_* ∼ N(0, $\sigma_{b}^{2\;}$), and *ε_ijkl_* is the residual error, with *ε_ijkl_* ∼ N(0, $\sigma_{\varepsilon }^{2\;}$).

The models were modified for specific cases based on data type: count data (PN) were analyzed using Poisson or negative binomial GLMMs when overdispersion was detected (overdispersion ratio > 1.5), ratio data (NSPP, LR, and NDVI) were handled with beta regression or log transformation, time-to-event data (NDVI decline, CC_50_ and CC_100_) were log-transformed, and continuous data were analyzed with linear mixed models. For traits with factors having only 1 or 2 levels, the corresponding terms were removed from the model. For cases where convergence issues occurred, the fixed-effects linear model was


$$\begin{array}{rl} Y_{ijkl} & =\mu +L_{i}+\;S_{j}+LS_{k}+(L\times S{)}_{ij}+(L\times LS{)}_{ik}\\ & \;+(S\times LS{)}_{\;jk}+(L\times S\times LS{)}_{ijk}+\varepsilon_{ijkl} \end{array}$$

where the random effect term *b_kl_* was omitted.

Each trait's data was standardized to z-scores to enable comparison of effect sizes across traits. Statistical significance of fixed effects in the mixed-effects model was assessed using ANOVA, with Satterthwaite's approximation for degree of freedom to compute *P*-values. For fixed-effects linear models, standard ANOVA was applied. Effect sizes (fixed-effect coefficients) and their 95% confidence intervals were extracted for both model types, providing estimates of the magnitude and direction of each factor's influence.

### PCA and RF regression

Following the mixed-effect modeling, a series of additional statistical analyses were conducted to investigate the relationships among the measured traits and their contributions to grain yield and peak LAI. These analyses included PCA and RF, each implemented using specific R packages and tailored to address distinct aspects of the data. PCA and RF analyses were carried out on global dataset rather than by separate treatment combinations. This decision was supported by Permutational Multivariate Analysis of Variance (PERMANOVA) using the *vegan* R package (version 2.6.10; Oksanen et al. 2022). While treatment main effects were statistically significant, interaction effect explained minimal variation in trait relationships (<0.4% for all interaction terms), indicating that the underlying multivariate structure remained consistent across experimental conditions.

PCA was performed on traits consistently available across all experimental combinations, excluding variables with sparse data. For the variables retained for the analysis, missing values were imputed using the *mice* R package (version 3.17.0; van Buuren and Groothuis-Oudshoorn 2011) with the predictive mean matching method (PMM), which leverages observed data patterns to estimate plausible replacements. The resulting dataset was standardized (mean = 0, standard deviation = 1) to ensure comparability across traits with differing scales. PCA was conducted using the *FactoMineR* package (version 2.11; Lê et al. 2008), employing singular value decomposition to extract principal components. Results were visualized with the *factoextra* R package (Kassambara and Mundt 2020).

Next, RF regression was applied as a complementary approach to the multivariate analyses specifically to identify and rank the relative importance of predictor variables influencing our primary outcome measures, grain yield and peak LAI. Separate models were constructed for each of the 2 response variables using the *randomForest* R package (version 4.7.1.2; Liaw and Wiener 2002), with 500 trees grown per model to ensure stability in predictions. Variable importance was assessed using 2 metrics: (i) the percent increase in mean squared error (%IncMSE), which quantifies the increase in prediction error when a given predictor is permuted, reflecting its contribution to model accuracy, and (ii) node purity, measured as the decrease in residual sum of squares (IncNodePurity), indicating a predictor's role in partitioning the data into homogeneous subsets within the trees.

### Accession numbers

The *GmJAG1* gene corresponds to locus Glyma.20G116200 (Wm82.a2.v1). The *GmJAG1* sequences in PI 612713A and PI 547745 were confirmed to carry the previously characterized *ln* allele (Jeong et al. 2012).

## Supplementary Material

kiaf663_Supplementary_Data

## Data Availability

The data underlying this article will be shared on reasonable request to the corresponding author.

## Supplementary material

The following materials are available in the online version of this article.

**Supplementary Figure S1.** Allelic discrimination of soybean lines using KASP marker analysis.

**Supplementary Figure S2.** Characterization of parental lines and analysis of donor parent genetic effects on measured traits.

**Supplementary Figure S3.** Summary statistics of morphological, physiological and agronomic traits measured across all experimental conditions.

**Supplementary Figure S4.** A-C_i_ response curves at different locations and row spacings.

**Supplementary Figure S5.** Temporal dynamics of canopy development traits.

**Supplementary Figure S6.** Aerial orthomosaic images of experimental plots at two field locations with contrasting row spacing treatments.

**Supplementary Figure S7.** Relative Efficiency (RE) analysis of Type II Modified Augmented Design.

**Supplementary Figure S8.** Yield comparison between leaf morphologies and model assessment of Peak LAI vs. Yield relationship.

**Supplementary Figure S9.** Multivariate analysis of experimental factors and traits.

**Supplementary Figure S10.** Model performance comparison across multiple canopy traits.

## Dive Curated Terms

The following phenotypic, genotypic, and functional terms are of significance to the work described in this paper:

PMM Gramene: AT2G45790
PMM Araport: AT2G45790

leaf AmiGo: PO:0025034

NIR Gramene: ostta10g00930
NIR Araport: ostta10g00930
