# Supplementary material for: Bigger is not always better: Optimizing leaf area index with narrow-leaf shape in soybean
Source: Plant Physiol. 2025 Dec 26;200(3):kiaf663. doi: 10.1093/plphys/kiaf663 (PMC13017493; doi:10.1093/plphys/kiaf663)
Supplement: kiaf663_Supplementary_Data [file kiaf663_supplementary_data.pdf]

A

Primer ID	Sequence
GmJAG1_F_HEX	GAAGGTGACCAAGTTCATGCTGACCAGAACGAAACCCCTTAG
GmJAG1_F_FAM	GAAGGTCGGAGTCAACGGATTGACCAGAACGAAACCCCTTAC
GmJAG1_R	TTCGAGGACTTGTTTGCCAT

B

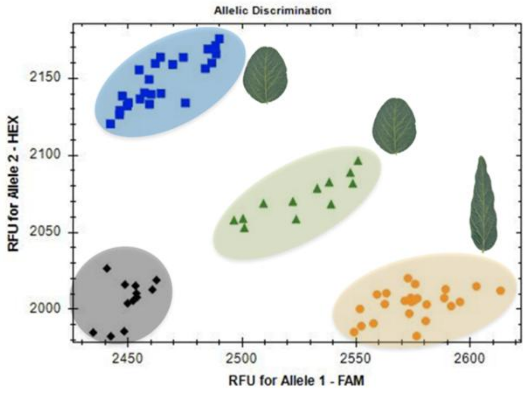

Supplementary Figure S1. Allelic discrimination of soybean lines using KASP marker analysis. A) Table listing the sequences of forward (F) and reverse (R) primers used in the GmJAG1 KASP marker analysis. The allele-specific ends are highlighted in red that differentiates between the two JAG1 allele types. **B)** Scatter plot illustrating allelic discrimination of segregating soybean lines based on a Kompetitive Allele Specific PCR (KASP) marker designed for a single nucleotide polymorphism (SNP) in GmJAG1. The x-axis represents Relative Fluorescence Units (RFU) for Allele 1 (FAM) and the y-axis represents RFU for Allele 2 (HEX). Blue squares indicate homozygous dominant broad-leaved lines, orange circles represent homozygous recessive narrow-leaved lines, green triangles denote heterozygous broad-leaved lines, and black diamonds signify no-DNA-template controls. Images of broad and narrow soybean leaves are included adjacent to the corresponding blue, green and orange data points to visually distinguish leaf morphologies.

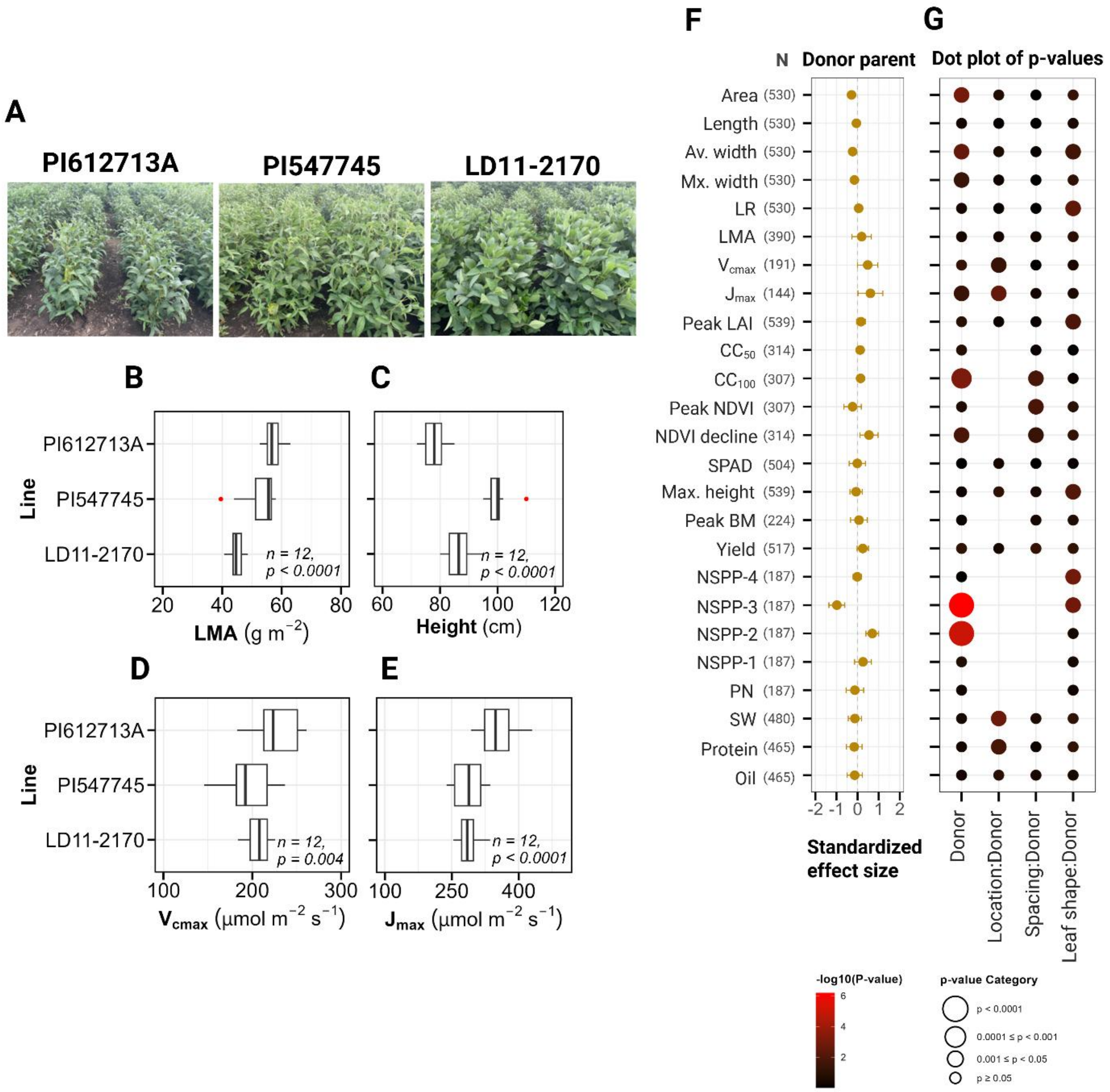

Supplementary Figure S2. Characterization of parental lines and analysis of donor parent genetic effects on measured traits. A) Field images of the three parental genotypes used in developing the isogenic lines, photographed during the 2022 growing season. The narrow-leaved donor parents PI612713A (top) and PI547745 (middle) display distinctly different leaf morphology compared to the broad-leaved recurrent parent LD11-2170 (bottom). **B-E)** Box and whisker plots comparing key physiological traits across the three parental lines: Leaf Mass per Area (LMA, B), Plant height at maturity (C), maximum rate of Rubisco carboxylation (V_{cmax}, D), and maximum rate of electron transport (J_{max}, E). For each trait, sample size (n) and p-values from ANOVA tests are displayed within each panel. **F)** Standardized effect sizes with 95% confidence intervals (orange dots and horizontal lines) for the Donor Parent factor (PI612713A as reference) across all 25 measured traits. Sample sizes are indicated for each trait. **G)** Significance of Donor Parent and its interactions with Location, Spacing and Leaf Shape from mixed-effects modeling. The size of dots indicates significance level categories, while color intensity represents $-\log_{10}$(p-value). All effects are relative to the reference donor parent (PI612713A).

Traits	Abbreviation (units)	Location: SoyFACE				Location: Energy FARM			
		Spacing: 38 cm		Spacing: 76 cm		Spacing: 38 cm		Spacing: 76 cm	
		Broad	Narrow	Broad	Narrow	Broad	Narrow	Broad	Narrow
**Leaf morpho-structural**									
Leaf area	Area (cm^2)	49 ± 6	35 ± 4	45 ± 6	31 ± 4	75 ± 9	53 ± 7	71 ± 12	51 ± 9
Leaf length	Length (cm)	9 ± 0.6	11 ± 0.7	9 ± 0.7	10 ± 0.8	12 ± 0.8	14 ± 1	12 ± 1	13 ±1
Leaf average width	Av. width (cm)	5 ± 0.6	3 ± 0.2	5 ± 0.5	3 ± 0.3	6 ± 0.6	4 ± 0.4	6 ± 0.7	4 ± 0.4
Leaf maximum width	Mx. width (cm)	7 ± 0.7	5 ± 0.3	7 ± 0.6	4 ± 0.5	9 ± 0.8	5 ± 0.5	9 ± 0.9	5 ± 0.6
Leaf ratio	LR (cm cm^{-1})	1.2 ± 0.1	2.4 ± 0.2	1.2 ± 0.2	2.3 ± 0.2	1.4 ± 0.2	2.5 ± 0.2	1.4 ± 0.2	2.5 ± 0.3
Leaf mass per unit area	LMA (g m^{-2})	43 ± 5	47 ± 5	48 ± 6	51 ± 7	43 ± 5	46 ± 6	47 ± 8	50 ± 8
**Leaf physiological**									
Max. rate of Rubisco activity	V_{cmax} (μmol m^{-2} s^{-1})	153 ± 15	155 ± 22	151 ± 18	158 ± 15	132 ± 16	131 ± 19	129 ± 18	134 ± 21
Max. rate of RuBP regeneration	J_{max} (μmol m^{-2} s^{-1})	201 ± 17	207 ± 26	202 ± 19	217 ± 25	173 ± 22	181 ± 29	193 ± 23	195 ± 31
**Canopy traits**									
Leaf area index	Peak LAI (m^2 m^{-2})	10.5 ± 0.8	9.1 ± 0.7	8.1 ± 0.9	7 ± 0.9	11.4 ± 0.7	10.5 ± 0.9	10.2 ± 1.2	8.7 ± 1.3
Days to 50% canopy coverage	CC_{50}	25 ± 2	26 ± 2	37 ± 3	39 ± 3	-	-	-	-
Days to 100% canopy coverage	CC_{100}	43 ± 3	44 ± 3	65 ± 2	66 ± 1	-	-	-	-
NDVI	Peak NDVI	0.94 ± 0	0.94 ± 0	0.94 ± 0	0.93 ± 0	-	-	-	-
Days to NDVI decline	NDVI decline	79 ± 1	79 ± 2	79 ± 2	79 ± 2	-	-	-	-
Relative greenness	SPAD	45 ± 2	47 ± 5	46 ± 2	47 ± 2	45 ± 3	46 ± 3	44 ± 2	46 ± 2
Plant height	Max. height (cm)	109 ± 2	107 ± 3	104 ± 3	102 ± 4	112 ± 5	110 ± 6	109 ± 4	106 ± 5
Digital Biomass	Peak BM (m^3 m^{-2})	-	-	-	-	6.2 ± 0.4	6 ± 0.4	5.7 ± 0.5	5.5 ± 0.6
**Yield and components**									
Grain yield	Yield (1,000 kg ha^{-1})	5.8 ± 0.4	5.7 ± 0.4	5 ± 0.5	4.9 ± 0.6	6.5 ± 0.7	6.5 ± 0.8	5.9 ± 0.6	6.0 ± 0.6
Proportion of 4-seeded pods	NSPP-4 (%)	1.8 ± 2.5	34 ± 13	-	-	-	-	-	-
Proportion of 3-seeded pods	NSPP-3 (%)	58 ± 17	51 ± 10	-	-	-	-	-	-
Proportion of 2-seeded pods	NSPP-2 (%)	37 ± 16	14 ± 15	-	-	-	-	-	-
Proportion of 1-seeded pods	NSPP-1 (%)	3 ± 4	1 ± 2	-	-	-	-	-	-
Pod numbers per plant	PN	40 ± 11	39 ± 8	-	-	-	-	-	-
100-seed weight	SW (g)	16 ± 1	15 ± 1	16 ± 1	15 ± 1	17 ± 1	16 ± 1	17 ± 1	15 ± 1
Protein content	Protein (% dry basis)	40.5 ± 0.9	40.1 ± 1.0	39.7 ± 1.0	39.4 ± 1.0	40.7 ± 0.8	40 ± 0.9	40.4 ± 1.3	39.6 ± 1.5
Oil content	Oil (% dry basis)	21.1 ± 0.6	21.1 ± 0.6	21.5 ± 0.7	21.3 ± 0.8	20.4 ± 0.5	20.6 ± 0.5	21 ± 0.5	20.9 ± 0.7

Supplementary Figure S3. Summary statistics of morphological, physiological and agronomic traits measured across all experimental conditions. This table presents the complete set of traits assessed in this study, including standardized abbreviations, measurement units, and mean values (± SD) aggregated across both locations (SoyFACE, Energy Farm), row spacings (38-cm, 76-cm) and leaf shape (broad, narrow). These summary statistics represent data from 204 isogenic lines evaluated across 600 experimental plots in the growing season of 2024.

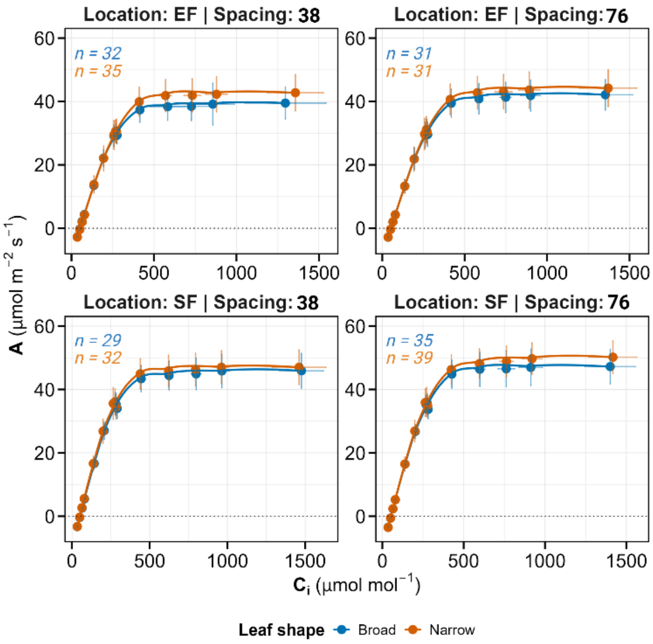

Supplementary Figure S4. A-C_i response curves at different locations and row spacings. The four panels show the relationship between intercellular CO_2 concentration (C_i) and photosynthetic rate (A) for broad- and narrow-leaved lines. Each data point represents the mean value with standard deviation bars in both axes. Fitted curves illustrate the photosynthetic response patterns for each leaf morphology.

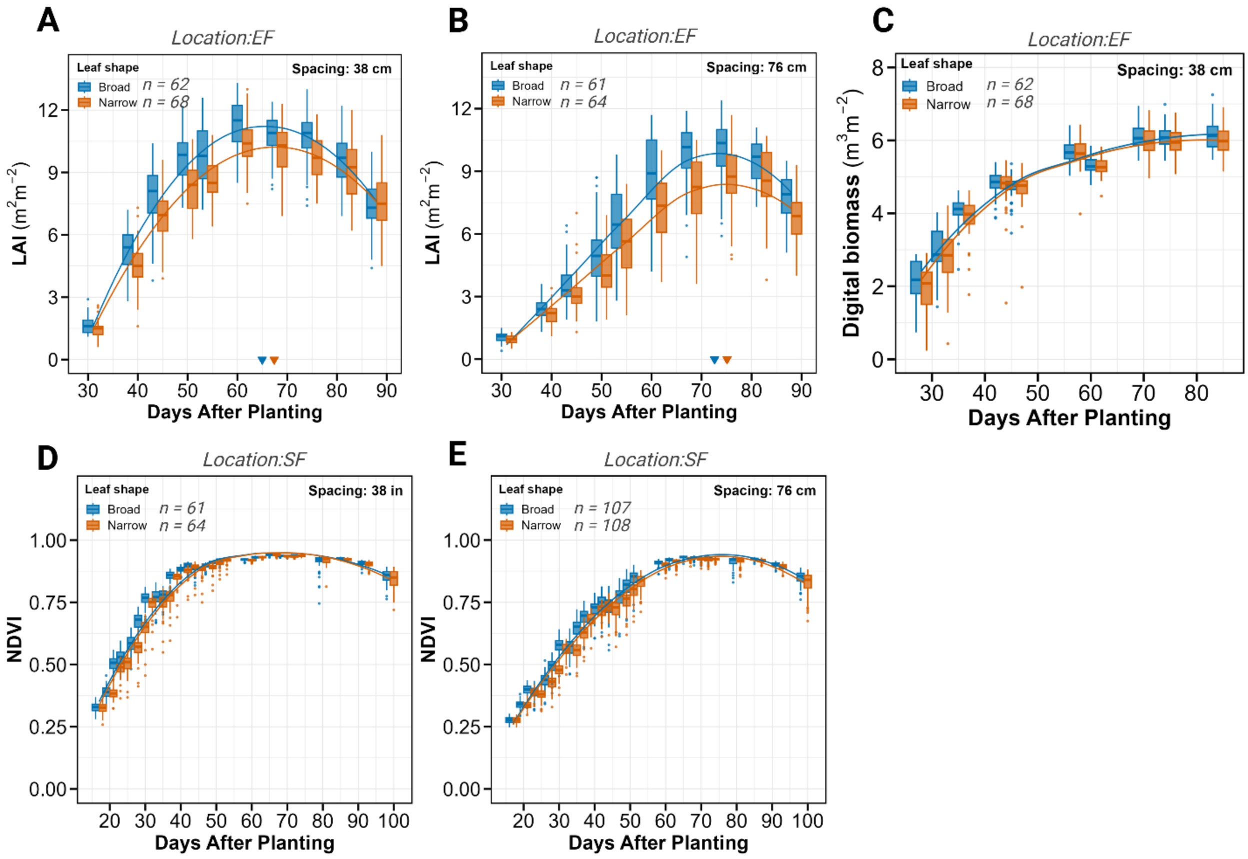

Supplementary Figure S5. Temporal dynamics of canopy development traits. A-B) Leaf Area Index (LAI) time course at Energy Farm at 38-cm and 76-cm row spacings. Each time-point displays box and whisker plots for broad- (blue) and narrow-leaved (orange) lines. Box plots display the median, interquartile range (IQR), and whiskers represent 1.5 times IQR with outliers shown as individual points. LOWESS curves illustrate the temporal trends for each leaf morphology. Triangles on the x-axis indicate model-predicted peak LAI values. **C)** Digital biomass progression over time at the Energy Farm at 38-cm row spacing. Data visualization follows the same format as panels A-B, with box and whisker plots for broad and narrow-leaved lines at each time point, fitted with LOWESS curves. **D-E)** Normalized Difference in Vegetation Index (NDVI) time course at SoyFACE in 38-cm and 76-cm row spacings. Data presentation follows the same format as panel C, showing the temporal dynamics of NDVI for both leaf morphologies.

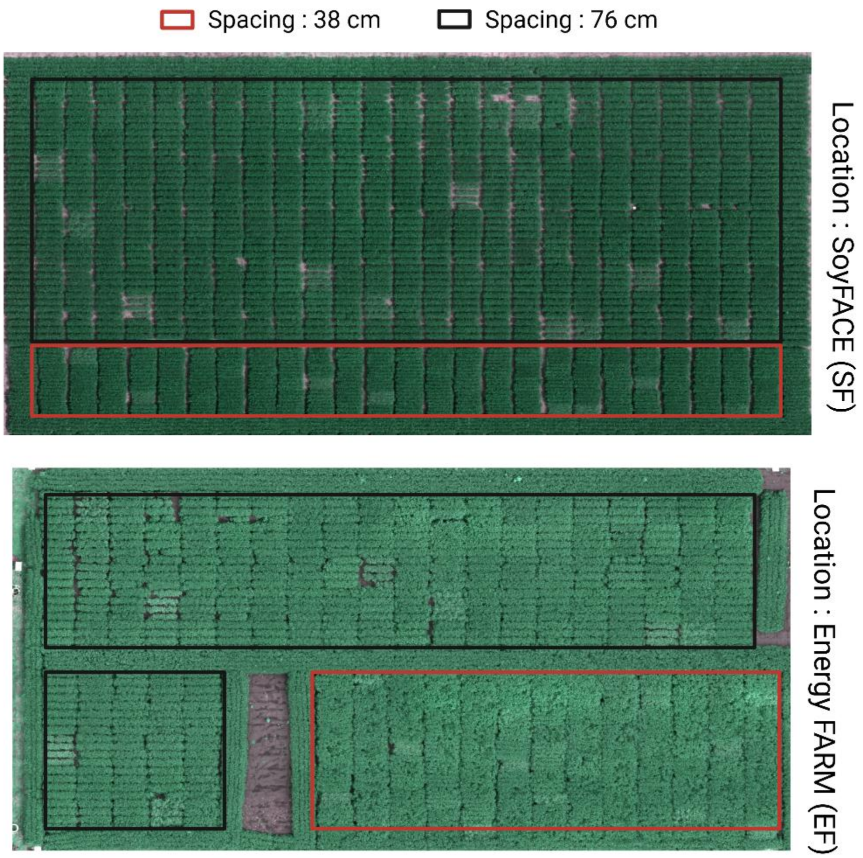

Supplementary Figure S6. Aerial orthomosaic images of experimental plots at two field locations with contrasting row spacing treatments. Top) SoyFACE facility, with the 76-cm row spacing treatment demarcated by black rectangular boundaries and 38-cm row spacing by red boundaries. **Bottom)** Energy Farm facility, located approximately 2 miles east from the SoyFACE site, with identical spacing treatments highlighted using the same color scheme. Images were captured approximately 75 days after planting when canopy closure had been achieved in most plots. Multispectral drone imagery captured during the growing season of 2024 spatial arrangement of soybean isogenic line trials.

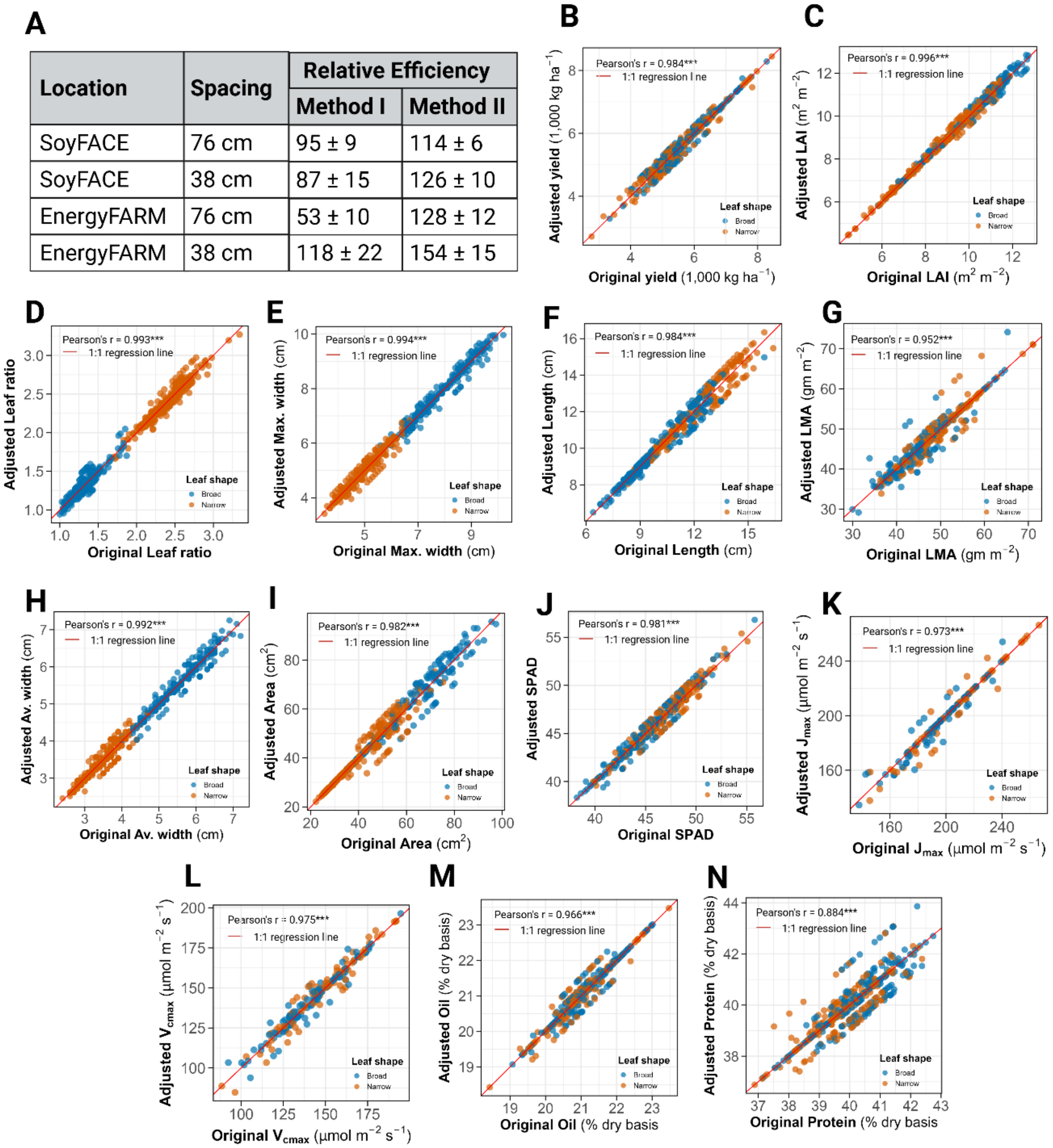

Location	Spacing	Relative Efficiency	
		Method I	Method II
SoyFACE	76 cm	95 ± 9	114 ± 6
SoyFACE	38 cm	87 ± 15	126 ± 10
EnergyFARM	76 cm	53 ± 10	128 ± 12
EnergyFARM	38 cm	118 ± 22	154 ± 15

Supplementary Figure S7. Relative Efficiency (RE) analysis of Type II Modified Augmented Design. A) Relative efficiency comparison between Method I and Method II across two locations and two spacings. Values represent the mean RE ± standard deviation calculated across all evaluated traits. **B-N)** Correlation between original and adjusted values for the traits which required adjustments. Data points are categorized by leaf shape, Broad (blue) and Narrow (orange). The red line represents the line 1:1 regression line. Pearson's correlation coefficients (r) are indicated with significance levels: * $p < 0.05$, ** $p < 0.01$, *** $p < 0.001$.

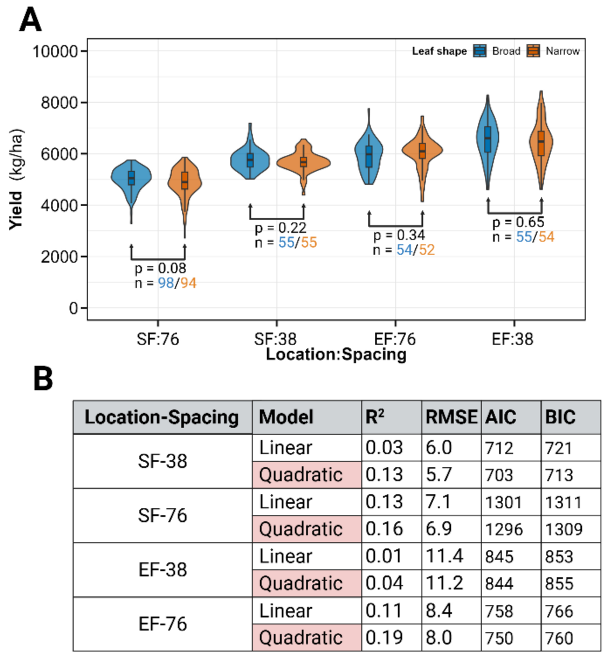

B

Location-Spacing	Model	R^2	RMSE	AIC	BIC
SF-38	Linear	0.03	6.0	712	721
	Quadratic	0.13	5.7	703	713
SF-76	Linear	0.13	7.1	1301	1311
	Quadratic	0.16	6.9	1296	1309
EF-38	Linear	0.01	11.4	845	853
	Quadratic	0.04	11.2	844	855
EF-76	Linear	0.11	8.4	758	766
	Quadratic	0.19	8.0	750	760

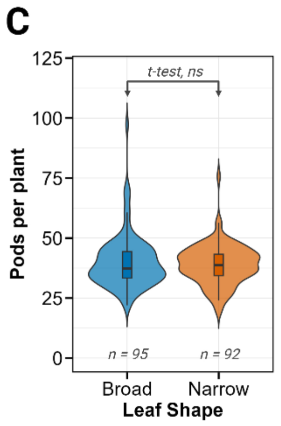

Supplementary Figure S8. Yield comparison between leaf morphologies and model assessment of Peak LAI vs. Yield relationship. A) Violin plots comparing yield performance between broad-leaved and narrow-leaved soybean isogenic lines across four locations and spacing combinations. The embedded box plots display the median, interquartile range (IQR), and whiskers represent 1.5 times IQR. Statistical significance was assessed using t-tests, with corresponding p-values and sample sizes (n) indicated for each comparison. **B)** Table summarizing the linear and quadratic regression model fits for the relationship between Peak LAI and Yield across the same four location and spacing combinations. For each model, R-squared (R^2), Root Mean Squared Error (RMSE), Akaike Information Criterion (AIC) and Bayesian Information Criterion (BIC) are reported to evaluate model performance, with the quadratic model providing the estimated Peak LAI at maximum yield. **C)** Violin plot comparing pod number per plant between broad (blue) and narrow (orange) leaf shapes, with sample size (n) and t-test p-value indicated, Data collected only at SoyFACE 76-cm row spacing.

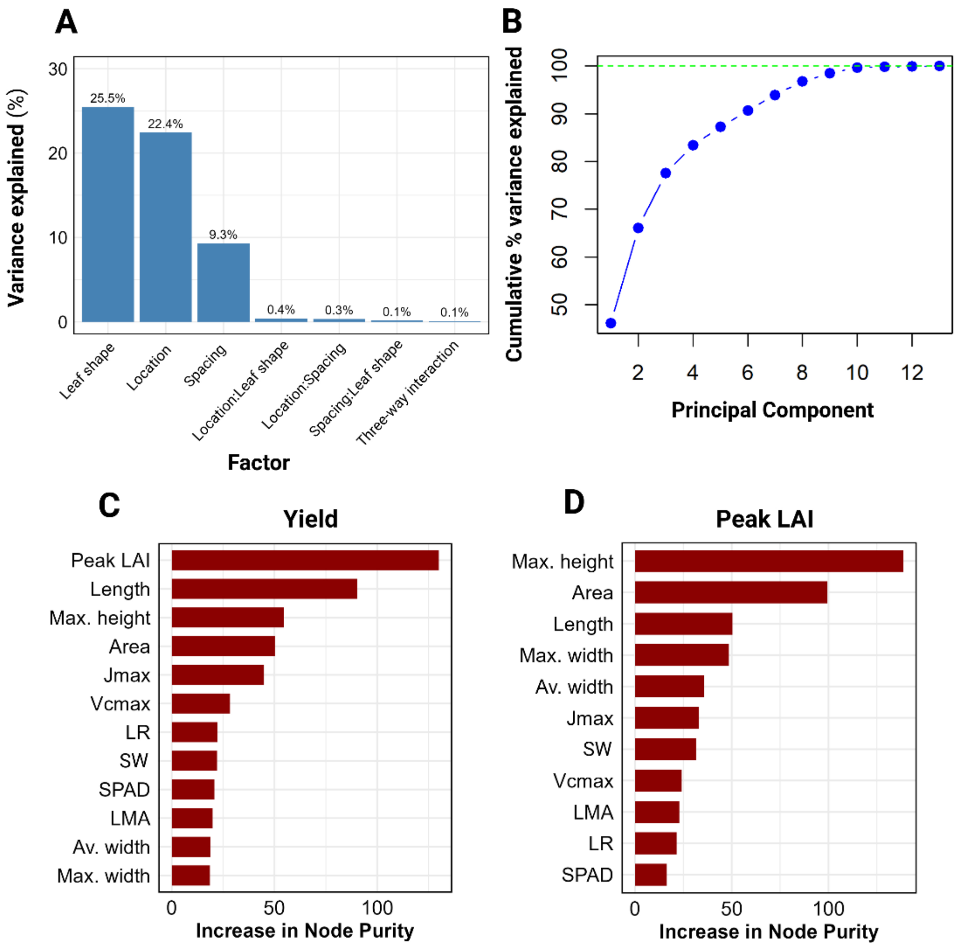

Supplementary Figure S9. Multivariate analysis of experimental factors and traits. A) Permutational Multivariate Analysis of Variance (PERMANOVA) results showing the variance explained by the three experimental factors: Location, Spacing and Leaf shape and their interactions. Blue bars represent the percentage of variance explained by each factor, with values displayed at the top of each bar. **B)** Cumulative variance explained by principal components, illustrating the contribution of each additional principal component to the total explained variance in the dataset. **C-D)** Random Forest Variable Importance analysis for Yield and Peak LAI. Red bars represent the increase in node purity for each predictor variable, indicating their relative importance in predicting the response variables.

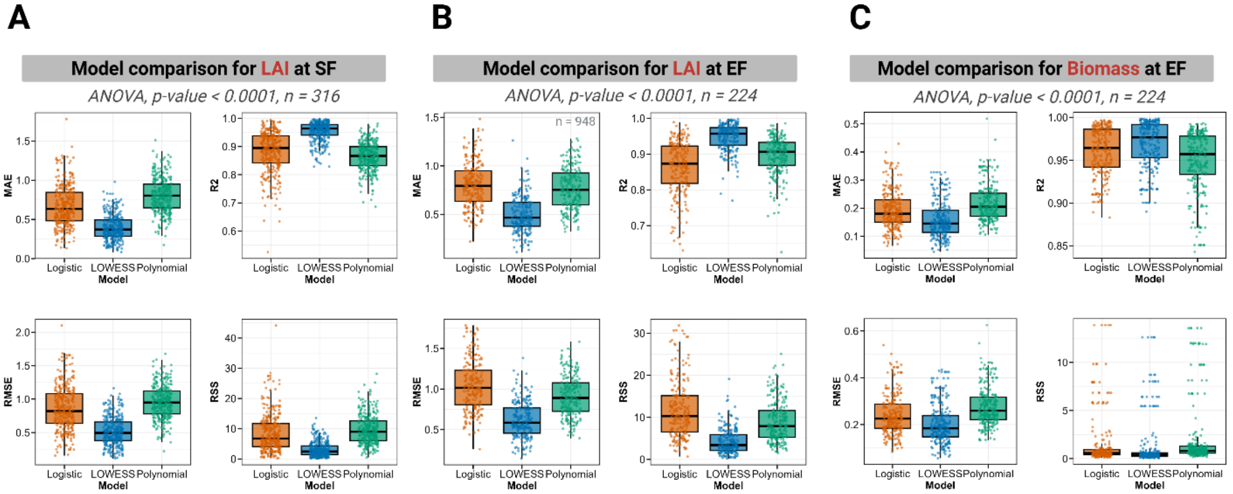

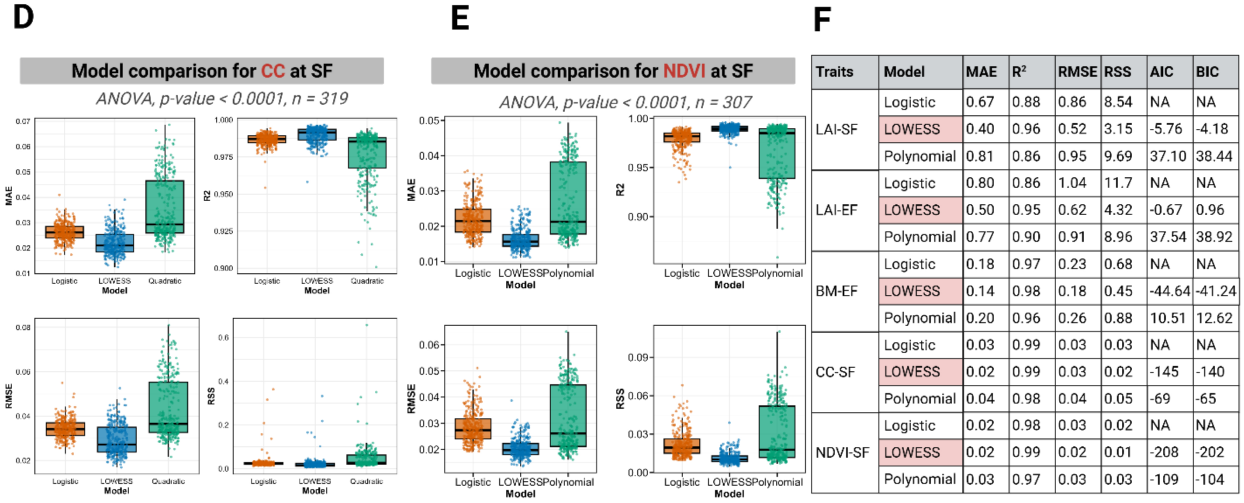

F

Traits	Model	MAE	R^2	RMSE	RSS	AIC	BIC
LAI-SF	Logistic	0.67	0.88	0.86	8.54	NA	NA
	LOWESS	0.40	0.96	0.52	3.15	-5.76	-4.18
	Polynomial	0.81	0.86	0.95	9.69	37.10	38.44
LAI-EF	Logistic	0.80	0.86	1.04	11.7	NA	NA
	LOWESS	0.50	0.95	0.62	4.32	-0.67	0.96
	Polynomial	0.77	0.90	0.91	8.96	37.54	38.92
BM-EF	Logistic	0.18	0.97	0.23	0.68	NA	NA
	LOWESS	0.14	0.98	0.18	0.45	-44.64	-41.24
	Polynomial	0.20	0.96	0.26	0.88	10.51	12.62
CC-SF	Logistic	0.03	0.99	0.03	0.03	NA	NA
	LOWESS	0.02	0.99	0.03	0.02	-145	-140
	Polynomial	0.04	0.98	0.04	0.05	-69	-65
NDVI-SF	Logistic	0.02	0.98	0.03	0.02	NA	NA
	LOWESS	0.02	0.99	0.02	0.01	-208	-202
	Polynomial	0.03	0.97	0.03	0.03	-109	-104

Supplementary Figure S10. Model performance comparison across multiple canopy traits. Box and whisker plots comparing model fit metrics (MAE, R^2, RMSE and RSS) for three curve-fitting approaches: Logistic (orange), LOWESS (blue) and Polynomial (green). Individual data-points represent experimental units. ANOVA p-values and sample sizes (n) are provided for each comparison. **A-B)** Model performance metrics for Leaf Area Index at SoyFACE and Energy Farm, respectively. **C)** Model performance metrics for Digital Biomass at Energy Farm. **D)** Model performance metrics for Canopy Coverage (CC) at SoyFACE. **E)** Model performance metrics for NDVI at SoyFACE. **F)** Summary table of mean values for traditional fit metrics (MAE, R^2, RMSE, RSS) and information criteria (AIC, BIC) across all assessed traits and models. Values represent means across all experimental units, combining both row spacings. Lower values of MAE, RMSE, RSS, AIC and BIC indicate better model performance, while higher R^2 values indicate better model fit.
